# Green Extraction and Fractionation of Chestnut Wood Waste: A Sustainable Pathway to Biopolymers and Antimicrobial Solutions

**DOI:** 10.1002/cssc.202500498

**Published:** 2025-07-31

**Authors:** Clelia Aimone, Giorgio Capaldi, Salah Chaji, Emanuela Calcio Gaudino, Anastasia Anceschi, Alessia Patrucco, Silvia Bonetta, Manuela Macrì, Giorgio Grillo, Giancarlo Cravotto

**Affiliations:** ^1^ Department of Drug Science and Technology University of Turin Via P. Giuria 9 10125 Turin (TO) Italy; ^2^ Institute of Intelligent Industrial Technologies and Systems for Advanced Manufacturing (STIIMA) Italian National Research Council (CNR) Corso G. Pella 16 13900 Biella (BI) Italy; ^3^ Department of Life Sciences and Systems Biology University of Turin Via Accademia Albertina 13 10123 Torino Italy

**Keywords:** biopolymers, chestnut wood waste, fractionation, green extraction, tannins

## Abstract

The disposal of agri‐food biomass waste, such as chestnut wood waste (CWW), poses significant environmental and industrial challenges, contributing to resource depletion and waste accumulation. The development of sustainable strategies for biomass valorization is crucial for reducing waste and promoting a circular economy. In this study, microwave‐assisted subcritical water extraction (MASWE) is investigated as an efficient and environmentally friendly method for the extraction of high‐value bioactive compounds such as condensed tannins (CTs), hydrolyzable tannins (HTs), and low‐weight polyphenols from CWW. The extraction process is followed by sequential membrane filtration and resin purification, adhering to green extraction principles to maximize yield and avoid water wastage. Furthermore, tannins are utilized in the synthesis of biopolymers, offering a promising strategy for developing novel materials with tailored properties. Their antimicrobial activity and enzyme‐inhibiting properties improve biopolymer formulations, unlocking diverse applications in sustainable materials. This approach through advanced extraction and fractionation protocols not only enhances biomass valorization—aligning with the circular economy in agri‐food waste management—but also supports advancements in green technology and the development of eco‐friendly materials.

## Graphical abstract

A sustainable valorization of chestnut wood waste is proposed through microwave‐assisted subcritical water extraction, optimized via central composite design. The extract is fractionated by membrane filtration and resin purification, enabling water recovery (up to 89%) and obtaining a tannin‐rich fraction, used to synthesize a PVA‐based biopolymer. Antioxidant, enzymatic, and antibacterial activities are investigated for the obtained fractions.

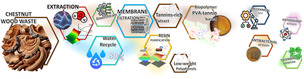

## 1 Introduction

Residues from agriculture and the food supply chain have traditionally been seen as waste that needs to be disposed of, resulting in significant environmental and economic consequences. Over the past two decades, there has been a growing shift in both research and industry towards more sustainable approaches to dealing with these by‐products. This shift aligns perfectly with the circular economy paradigm, which emphasizes resource reuse, reducing the depletion of raw materials and minimizing waste production.^[^ ^1^, ^2^ ^]^

As a result, production leftovers are now increasingly regarded as valuable sources of bioactive compounds and other high‐value constituents.^[^ ^3^ ^]^ Residual bioactive compounds in biomass can be recovered and valorized across various industries, including food, cosmetics, pharmaceuticals, and materials.^[^ ^4^ ^]^ The main goal of the so‐called green extraction is to obtain high‐added‐value products from biomass—preferably from waste—through inherently safer and environmentally friendly methods, embodying the concept of “benign‐by‐design”.^[^ ^5^, ^6^ ^]^ This is accomplished by extraction intensification, which minimizes the use of organic solvents and chemicals, reduces process by‐products, and enhances time and energy efficiency. Among high‐potential biomasses, chestnut wood (CW) stands out as a rich source of bioactive compounds that can be extracted and valorized. The chestnut tree (*Castanea sativa*, Mill.), is a deciduous plant belonging to the Fagaceae family. Its distribution area ranges from southern Europe and North Africa to northwestern Europe and eastward to western Asia. Regarding Europe, chestnuts cover an area of more than 2.5 million ha, most of which (89%) is concentrated in France and Italy, followed by Spain. The chestnut tree, cultivated to produce timber and fruits, is rich in bioactive compounds, widely extracted and used in different applications.^[^ ^7^, ^8^, ^9^ ^]^ Apart from the common uses, CW is particularly valued for its polyphenol content, including phenolic acids and tannins. This latter class of bioactive compounds could be categorized into condensed tannins (CTs), which consist of flavonoid units arranged in a polymeric structure, and hydrolyzable tannins (HTs), where sugar moieties are bound to ellagic and gallic acid units via ester bonds, giving rise to ellagitannins and gallotannins. It is worth noting that, CW waste (CWW), a by‐product of timber production and tree pruning, retains a higher amount of these and other bioactive compounds, including polyphenols, known primarily for their antioxidant,^[^ ^10^ ^]^ anti‐inflammatory,^[^ ^11^ ^]^ antimicrobial,^[^ ^12^ ^]^ and anticancer properties.^[^ ^13^ ^]^ Moreover, the annual production of CWW is substantial and exploring strategies to recover valuable compounds from this by‐product represent an important challenge with promising potential. Recovering these bioactive compounds typically involves hot water maceration or organic solvents, alone or mixed with water. However, conventional methods are energy‐intensive and time‐consuming. To enhance efficiency, novel enabling technologies have been explored, focusing on reducing toxic solvents, extraction time, and energy consumption. Nowadays, the most exploited technologies in this field include microwave‐assisted extraction (MAE),^[^ ^14^ ^]^ ultrasound‐assisted extraction (UAE),^[^ ^15^ ^]^ pulsed electric fields (PEF),^[^ ^16^ ^]^ cavitation,^[^ ^17^ ^]^ supercritical fluid extraction,^[^ ^18^ ^]^ enzyme‐assisted extraction, high‐pressure homogenization,^[^ ^19^ ^]^ and deep eutectic solvents extraction,^[^ ^20^ ^]^ among others. Besides the extraction of natural compounds, the downstream processing also plays a crucial role in the protocol optimization, particularly in reducing costs and saving energy. In this regards, key technologies are membrane filtration (for fractionation and water recovery), resins purification, chromatography, and crystallization. In this study, the focus was the microwave‐assisted subcritical water extraction (MASWE) of CWW, followed by membrane sequential filtration and resin purification, trying to follow the pillars of green extraction to recover both added value Low‐weight polyphenols and tannins. Subcritical water extraction (SWE) is an innovative technique that uses water in its subcritical state (100–374 °C) under pressure to maintain its liquid form.^[^ ^21^ ^]^ In this state, water's polarity decreases, resembling certain organic solvents and enhancing its extraction efficiency. As a widely available, cost‐effective, and nonhazardous solvent, water dissolves polar compounds efficiently.^[^ ^22^ ^]^ Under subcritical conditions, its solubility becomes temperature‐dependent, allowing the extraction of moderately polar bioactives like polyphenols and tannins. This makes SWE a promising alternative for recovering valuable compounds from CWW. Notably, SWE conditions can also be achieved using a hybrid technique that exploits fast microwave (MW) heating under controlled temperature and pressure. In this approach, water is heated above its boiling point within sealed vessels, enabling efficient and precise extraction.

Despite extraction technology being the core of such a process, the purification steps of the extractives are equally crucial. When extracting in water, a vast plethora of compounds will be present in the aqueous medium alongside the target molecules, making their recovery and isolation essential to enhance selectivity. For the purification purposes, several technologies are currently available, with innovative ones aiming to overcome the drawbacks typical of conventional protocols. Certain purification techniques are expensive and typically not environmental‐friendly; thus, their application is usually limited to the isolation of high‐value compounds (such as pharmaceuticals) where the associated costs can be justified by the market value of the products. Membrane filtration systems have large diffusion in several industrial applications. This physicochemical technique separates compounds primarily by molecular weight, determined by their molecular weight cut‐off (MWCO), achieved through the pressure driven applications of semi‐permeable porous membranes. It is possible to list microfiltration (MF), ultrafiltration (UF), nanofiltration (NF), and reverse osmosis (RO). One of the main advantages of this technology is the low energy consumption compared to other separation methods, coupled with its capability to operate at room temperature (RT), crucial when dealing with thermally sensitive compounds such as polyphenols and other metabolites.^[^ ^23^ ^]^ Application of membrane filtration to polyphenolic rich extracts can facilitate the partial fractionation of targeted compounds, through size exclusion. Several studies in the literature report successful applications of these methods in this context.^[^ ^24^, ^25^, ^26^, ^27^ ^]^ Furthermore, the use of NF or RO allows for the recovery of water solutions with very low solute concentration, enabling the design of protocol that incorporate water reuse within the same process.

Another important approach for extract purification, which is cost‐effective and easily scalable at the industrial level, is the use of resins adsorption. Macroporous resins enable the adsorption of molecules, such as polyphenols, from aqueous solution through hydrophobic binding and aromatic stacking, depending on the nature of the resin used. The phytochemicals from crude extract adsorbed on the resin can be then recovered by organic bioderivable solvents, like ethanol or ethyl acetate. Since sugars and salts do not interact with this type of resins, they can be easily removed by water elution, thereby enhancing the selectivity of the purified extract.^[^ ^28^ ^]^ In recent years, there has been a growing interest in applying macroporous adsorption resins to purify polyphenols from plant materials.^[^ ^29^, ^30^, ^31^, ^32^ ^]^ This study aimed to enrich CWW tannins in MASWE extract using a membrane filtration cascade approach. Beside this main objective, adsorption resins were utilized to recover CWW polyphenols from a side‐product fraction obtained during the process, thereby enhancing the overall valorization of the treated biomass.

The studied protocol herein reported led to achieve a tannin‐rich fraction form CWW, not only investigating the MAE and the purification steps but also incorporating it in an innovative green biopolymer. This approach highlights the potential of tannin‐based materials in sustainable applications. Developing biopolymers from bioderived molecules alongside synthetic polymers (e.g., polyvinyl alcohol (PVA) and polylactic acid (PLA)) offers a sustainable alternative to petroleum‐based plastics.^[^ ^33^, ^34^ ^]^ Tannins enhance polymer properties and reduce environmental impact, making them valuable for applications such as active food packaging, biodegradable mulch, antiadhesive coating with antimicrobial properties,^[^ ^35^ ^]^ wood adhesives,^[^ ^36^ ^]^ and adsorbent for water pollutants.^[^ ^37^ ^]^ Their intrinsic antioxidant and antimicrobial properties further expand their potential. Combining tannins with biodegradable polymers is a key strategy for creating innovative, high‐performance materials. Additionally, the antimicrobial activity of tannins and polyphenols, along with their enzymatic inhibition properties, can be leveraged in biopolymers, unlocking a wide range of potential applications. Combining biomolecules—particularly tannins—with biodegradable polymers is one of the most effective strategies for developing novel materials with tailored properties.

This study seeks to develop a sustainable protocol for the extraction of tannin‐rich fraction from CWW, focusing on both extraction and purification while highlighting tannins’ potential in sustainable applications. By addressing waste biomass valorization—a key aspect of the circular economy in agri‐food waste—this research contributes to advancements in green technology and the development of eco‐friendly materials.

## 2 Experimental Section

Table of Abbreviations. Acronyms and abbreviations adopted throughout the manuscript are summarized in Table S1, Supporting Information.

### 2.1 Biomass Feedstock

The CW sawdust (CWW) used in this study is a residue produced during the mechanical processing of chestnut timber, particularly during sawing, trimming, and surface finishing operations carried out in sawmills and wood manufacturing facilities. CWW was gently provided by SilvaTEAM SpA (San Michele Mondovì, Italy). The particles size distribution is reported in Figure S1, Supporting Information. For the extraction process, the biomass was sieved and the fraction with a particle size <1000 μm was selected.

### 2.2 MASWE

During the screening for optimal conditions, the chosen amount of CWW (ranging from *approx*. 5 to 60 g) was mixed with 200 mL of water, according to the liquid/solid ratio (L/S) given from the central composite design (CCD, approx. ranging from 3 to 40). To hydrate the matrix the mixture was soaked for 5 min in a US bath (40 KHz, 200 W) in a 1 L Teflon vessel. The vessel was then introduced into a MW multimodal reactor (SynthWAVE, Milestone, Bergamo, Italy), able to work with external gas. After three N_2_ purge (to avoid oxidative degradation), the system was pressurized to reach subcritical conditions and avoid water ebullition at every explored temperature (10 bar). Cooling down is provided by a chiller operating at 8 °C. The samples were treated according to CCD experimental design (details in Table S2 and S3, Supporting Information) at different temperatures, (approx. ranging from 80 to 200 °C) and time (ranging from 2 to 60 min). The desired temperature was reached with a 6 min ramp with a maximum irradiation power of 1500 W. The magnetic stirring was set as 400 rpm and the releasing parameters standardized for all tests (T: <35 °C; pressure rate: 10 bar min^−1^). After the extraction, the resulting liquor was separated by centrifugation at 4200 rpm for 5 min (Cence Hunan Xiangyi Laboratory Instrument Development Co., Ltd., Changsha, China) and the supernatant filtered under vacuum. To recover the dry extract, an aliquot of the supernatant was freeze‐dried (−60 °C, 0.2 mbar, Telstar Lyotest, Azbil Telstar SL, Terrassa, Spain). The dried extracts have been characterized by means of dry yield, total polyphenolic content (TPC), and tannins/low‐weight polyphenol distribution (see the **Analytical Section**). The L/S ratio (10, 20, and 30 L/S) have been investigated on the most favorable identified conditions of temperature and time. The optimized parameters have been adopted for the following kinetic and downstream investigations. To achieve the required volume for the membrane processing, a scale‐up has been performed (30 g of CWW, 600 mL of deionized water), producing several batches.

### 2.3 Downstream Process

#### 2.3.1 Membrane Filtrations

Different sequential steps were carried out to fractionate the CWW extract with membranes. To avoid instrument clogging, a clarification was performed before the filtration. The solution to be processed was inserted in the tank and the retentate continuously recirculated inside the system until the desired volume of permeate was removed. The permeate was collected in a graduated 2 L cylinder, monitoring process time.

The system, composed by a pilot skid (PB100, Hydro Air Research Srl, Lodi, Italy), was equipped with different membranes: DKU 1812 named as NF_150_ (150–300 Da, 0.38 m^2^ filtering area, operating P: 10 bar), SDR68‐1812C named as NF_600_ (600–800 Da, 0.33 m^2^ filtering area, operating P: 2 bar), SDR10‐1812 membrane named as UF_1000_ (1000 Da, 0.33 m^2^ filtering area, operating P: 4 bar), SDR5‐1812 membrane named as UF_5000_ (5000 Da, 0.33 m^2^ filtering area, operating P: 4 bar).

**Figure 1:**
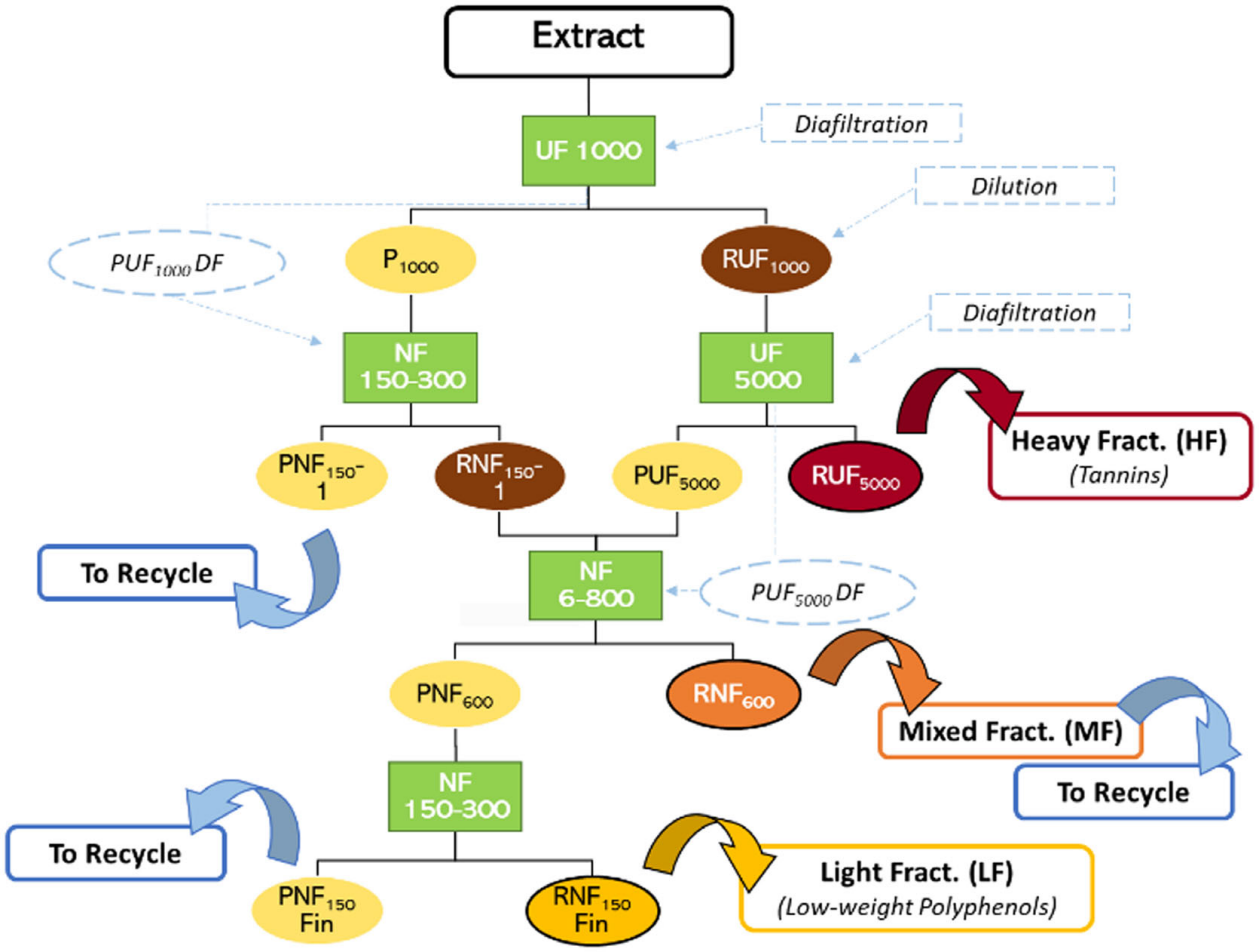
Membrane filtration scheme.

Detailed procedure is summarized in **Figure** **1** and in the Supporting Information (“*Membrane Filtration Detailed Procedure*”)

#### 2.3.2 Resin Purification

Resin purification was performed on the light fraction (LF) from the membrane process (Figure 1). A prescreening was carried out to identify the best type of resin, between XAD‐4, XAD‐7 HP, XAD‐16, and Sepabeads SP 825 L (SB) (Sigma‐Aldrich, St. Louis, MO, USA).

XAD‐4 is a polyaromatic adsorbent for small hydrophobic compounds, normally considered an excellent choice for the adsorption of organic substances of relatively low molecular weight, such as phenols. XAD‐7 HP is a crosslinked aliphatic acrylic adsorbent resin that adsorbs and releases ionic species through hydrophobic and polar interactions. Amberlite XAD‐16 is a polymeric adsorbent widely utilized for adsorption of organic substances from aqueous systems and polar solvents. Sepabeads 825 L is highly porous styrenic adsorbents. It has a large surface area and a narrow pore size distribution, and it offers high capacity for small molecules.

0.5 g of dry resin was mixed with 10 mL of aqueous solution of LF. The mixture was shaken vigorously (600 rpm) for 12 h at RT in an orbital shaker (Synthesis 1, Heidolph Instruments GmbH Schwabach, Germany). The mixture was then separated by centrifuge (4200 rpm for 5 min) and vacuum filtration of the supernatant. The resin was washed with 10 mL of fresh water and recovered. 10 m of EtOH was used for the desorption step, carried out at 600 rpm, for 8 h at RT. The mixture was then separated by centrifuge (4200 rpm for 5 min) and vacuum filtration of the supernatant. All the liquid fractions were analyzed by means of TPC determination (see the **Analytical Section**).

Using the best type of resin identified in the prescreening (Sepabeads SP 825 L), a CCD was conducted on both adsorption and desorption steps to optimize the process (details in Table S4 and S5, Supporting Information). The variables investigated were as follows. 1) *Adsorption*: temperature (ranging from 20 to 50 °C); extract (LF) to resin ratio (ranging from 10 mg_Extract_/g_Resin_ to 115 mg_Extract_/g_Resin_). Fixed amount of dry resin (0.5 g) and solution volume (10 mL). 2) *Desorption*: temperatures (ranging from 20 to 50 °C); solvent to resin ratio (ranging from 1.5 mL_Solvent_/g_Resin_ to 90 mL_Solvent_/g_Resin_). Fixed amount of dry resin (0.5 g) and adsorbed amount of extract (LF) (25 mg_Extract_/g_Resin_).

180 minutes was adopted as a screening time for both the studies, to allow the system to reach the steady state. The desorption tests were performed on the absorption optimized protocol.

#### 2.3.3 CCD

The experimental design was conducted using the Design‐Expert 13 software. Statistical tests were conducted using IBM SPSS Statistics for Windows, version 23.0 (IBM Corp., Armonk, NY, United States). A Tukey test was used for post hoc analysis at the 5% confidence level.

#### 2.3.4 Kinetic Description

After the CCD determination of best conditions, kinetic investigations were performed on both the extraction and the resin purification protocols (adsorption and desorption) to identify the optimal extraction and purification times.

Hereafter, the evaluated time frames together with the constant conditions: 1) *Extraction*—time screening: 0, 2, 5, 10, 20, and 60 min. Fixed parameters: temperature (176 °C), L/S ratio (20) and heating ramp (6 min); 2) *resin purification*—time screening: 0, 5, 10, 20, 60, and 180 min.

*Adsorption fixed parameters*: dry resin (0.5 g), temperature (25 °C), extract (LF) to resin ratio (25 mg_Extract_/g_Resin_).

*Desorption fixed parameters*: dry resin (0.5 g), temperature (20.86 °C), solvent to resin ratio (47.5 mL_Solvent_/g_Resin_).

The kinetic interpretation was conducted testing different mathematical models, namely pseudo‐first order (PFO), pseudo‐second order (PSO), Weber–Morris (parabolic or intraparticle diffusion model), Elovich, power law, and Peleg (hyperbolic).^[^ ^38^ ^]^ Experimental data are fitted by linearization and parameter extrapolated consequently. The exploited equations are reported in Table S6, Supporting Information.

### 2.4 Analytical Section

#### 2.4.1 Total Polyphenolic Compounds (TPC)

To quantify the TPC, the *Folin‐Ciocalteu* Assay has been performed.^[^ ^39^ ^]^ Dry extracts were dissolved in deionized water (in a range of concentration appropriate for the reading). In each test tube, 250 μL of sample solution (extract or water for the blank) was placed, along with 4 mL of deionized water, 500 μL of a solution of Na_2_CO_3_ (10% *w/v*), and 250 μL of a solution of *Folin‐Ciocalteu* (diluted 1:1). The obtained solution was vigorously shaken and then incubated at RT in the dark for 25 min. After that period, the absorbance at 725 nm was read, and the content in polyphenols was estimated by means of a calibration curve made with gallic acid, expressing the values as gallic acid equivalents (GAEs). Analyses were performed in triplicate, reporting results as average ± the standard deviation. Considering the TPC analysis of the biopolymer, a suitable weight of specimen is sampled in order to have a response in the linear range of the test. This aliquot has been suspended in 250 μL of deionized water, and all the other reactants were added in the same way and concentrations. The dark was maintained thanks to the aluminum foil, and for all the incubation time, the samples were maintained under constant stirring (650 rpm).

#### 2.4.2 Total Tannin Content

Tannin determination was performed by using Peri and Pompei protocol with some modifications and optimizations previously developed in other works.^[^ ^26^, ^40^ ^]^ Briefly, the analysis involves the reaction between a hemisulfate cinchonine solution (0.5% *w/v*) and the extract solution. After overnight incubation at 4 °C, the precipitation of the tannate cinchonine occurs. After ultracentrifugation, the supernatant consists of a polyphenol‐rich solution, while the precipitate represents the tannic fraction. The supernatant was separated and analyzed with the *Folin–Ciocalteu* test. The total tannin semi‐quantitative content was calculated by difference and expressed as GAE.

#### 2.4.3 HTs and CTs

The tannins’ fraction was also characterized in terms of HTs and CTs, as previously reported by Aimone et al.^[^ ^26^ ^]^ The precipitate formerly obtained from the reaction with cinchonine hemisulfate was resuspended with 600 μL of EtOH_aq_ 50%. Then, 500 μL of this suspension was mixed with 250 μL of H_2_O/HCl 36% (5:2 v/v) and 250 μL of an aqueous solution of formaldehyde 4.8%. The mixture was vigorously shaken and incubated overnight and then centrifuged at 26 000 rpm for 5 min at 10 °C. The supernatant, containing the hydrolyzable fraction, was analyzed by Folin–Ciocalteu. The precipitate contains CTs. The amount of this fraction was determined by the difference between the total and the hydrolyzable fraction.

#### 2.4.4 Total Sugar Content (TSC)

The TSC present in the samples was assessed using of a fast and simple colorimetric assay, known as anthrone method.^[^ ^41^ ^]^ The operative steps were carried out as previously described in other works.^[^ ^42^ ^]^ Briefly, the freshly prepared reagent (anthrone dissolved in concentrated sulfuric acid) is added to the sample solution and incubated at 100 °C for 20 min. The mixture was then cooled down to RT, and the absorbance is registered at 620 nm. Glucose was used to prepare a calibration curve, expressing the results as mg_Glu_/g_extract_. Analyses were performed in triplicate, reporting results as average ± the standard deviation.

#### 2.4.5 Antioxidant Capacity

The antioxidant capacity of the extracts was evaluated by using the stable free radical DPPH· (2,2‐diphenyl‐1‐picrilidrazile), as described by Brand‐Williams et al.^[^ ^43^ ^]^ The CW extract, reach in antioxidant compounds, cause the DPPH· radical inhibition, which is quantified by the decoloration of the solution (from violet to colorless). This event was monitored and referred to a Trolox methanolic solution, considered as a reference antioxidant. The IC_50_ (the extract concentration able to inhibit 50% the DPPH· radical at equilibrium) was evaluated as the scavenging activity metric. By operating subsequent dilutions, solutions of different concentration were analyzed and the absorbance was read at 515 nm (Cary 60 UV‐vis spectrophotometer, Agilent Technologies, Santa Clara, CA, USA). The Bobo Least Squares software (ver. 0.9.1.) was used to process the obtained absorbance data, to identify a fitting Probit regression. A blank containing only a hydroalcoholic solution (water and methanol) was used to zero the instrument; a blank sample containing the dry extract, without the DPPH· radical, was used to evaluate the matrix effect; and a reference sample, containing water and DPPH· radical, was used to normalize the results and verify the absorbance of the reactive. Considering the biopolymer analysis for antioxidant activity, two different experiments were conducted: surface and release activity. For the former one, the weighted specimen was suspended in 5 mL of MeOH, and 5 mL of DPPH· solution has been added. After the time of the assay, the solution has been analyzed. For the release test, the weighted specimen was suspended in 10 mL of MeOH and stirred for 20 min, simulating the conditions of the test. Then, the methanolic solution was removed and concentrated to 5 mL to be comparable with the previous test. An aliquot of the liquid tested by adding DPPH· solution in a 1:1 ratio.

#### 2.4.6 MS/MS Targeted Characterization of Signature Compounds

High‐resolution mass spectrometry (HRMS) and tandem mass spectrometry (MS/MS) experiments were performed using a Sciex ZenoTOF 6700 system equipped with a TurboIon Spray source, operated in both positive and negative electrospray ionization (ESI) modes. The system was controlled by the SCIEX OS software. Flow rate was set at 10 μl min^−1^ during the infusion. The TOF‐MS data were acquired over an *m/z* range of 100–1000 with an accumulation time of 0.25 s per scan. The ESI source was operated at 350 °C in positive ionization mode and 450 °C in negative ionization mode, with capillary voltages of 5500 and −4500 V, respectively, curtain gas was maintained at 45 psi. Declustering potential (DP) was set at 80 and −80 V and collision energy (CE) 10 e −10 V for positive and negative mode, respectively. For guided MRM‐HR experiment in infusion mode, the initial parameters matched those used in TOF‐MS acquisition. During the optimization process, DP and CE were systematically varied, ranging from −150 to +120 V (DP) and −70 to +110 V (CE). Samples, both standards (1 mg L^−1^) and extracts (2 mg mL^−1^), were dissolved in LC‐MS grade water or MeOH, filtered and diluted 1:1 with 0.1% formic acid prior the analysis.

### 2.5 Biopolymer Synthesis and Characterization

#### 2.5.1 Preparation

**Scheme 1:**
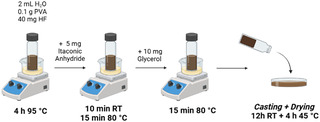
Polymers preparation and casting.

The biopolymer was prepared using PVA as the base polymer, loaded with the heavy fraction (HF) obtained via membrane filtration (Figure 1). The methodology was inspired to Liao et al.^[^ ^44^ ^]^ with some modification (see **Scheme** **1**): PVA (100 mg) and HF (40 mg) were dissolved in 2 mL of deionized water with the aid of sonication (40 kHz, 5 min, in an ultrasonic bath, Weber Ultrasonics, MG200TFDMF 40–80–120, Karlsbad, Germany). The resulting mixture was placed in a hot oil bath and maintained at 95 °C for 4 h under magnetic stirring. Subsequently, 5 mg of itaconic anhydride (ItA) was added, and the mixture was kept at RT for 10 min before being incubated at 80 °C for 30 min. Midway through this incubation period, 20 mg of glycerol was added as a plasticizer. At the end, the liquid was casted in a small Petri dish and dried for 12 h at RT and subsequently for 4 h at 45 °C. Several polymers were also prepared screening the relative quantity of HF, anhydride, or glycerol. A test was performed where 25% of PVA was replaced with a bioderived, pectin‐rich fraction (PF) extracted from orange peel waste. The extraction was performed by exploiting MASWE (120 °C; S/L 1:20; 10 min), followed by membrane UF 1000, based on a protocol previously optimized. The aim of this modification was to enhance the bioderived content of the polymer, promoting biomass valorization, and improving at the same time the material's mechanical properties.

#### 2.5.2 Soil Burial Test

To assess the biodegradability of the synthesized polymers, a trial has been conducted by burying a specimen of biopolymer into a pot filled with natural soil, basing on the procedure explained by Sen and Das, downscaled in dimensions.^[^ ^45^ ^]^ As a pot, aluminum cups have been used (diameter: 8 cm; height: 3.9 cm; capacity: 0.12 L) and half filled with soil. The polymer was placed between two gauzes to prevent soil from sticking, and this layer was positioned hallway up in the soil, covering the bandage cloth with soil. The test was performed on 4 different biopolymers, each in one different pot, and they were sprinkled with water on the surface (12 mL) and placed inside a sealed container with saturated NaCl water to maintain a constant humidity level.

The polymers were unearthed (after 1, 4, 8, 11, 15, 18, and 90 days), cleaned up with a soft brush to remove the even smallest particle that adhere to the surface and placed in a desiccator to remove humidity. After 2 h, the weight was measured and the decrease in % was calculated. After that, the polymers have been buried again in the same soil and sprayed again the same amount of water.

#### 2.5.3 Thermogravimetric Analysis (TGA)

To perform TGA, a TGA Mettler Toledo (Columbus, Ohio, USA) apparatus was used, with STARe system applied, under 100 mL min^−1^ N_2_ flow. On average, 10 mg of sample was placed in an alumina crucible and heated up to 800 °C, with a 10 °C min^−1^ ramp.

#### 2.5.4 Differential Scanning Calorimetry (DSC)

For the DSC analysis, the TA 3000 instrument from Mettler Toledo (Columbus, Ohio, USA) with a DSC 20 cell was used. The program operated within a temperature ranging from 30 to 220 °C, with a heating ramp of 10 °C min^−1^ under a nitrogen flow of 50 mL min^−1^. The analysis was performed over a temperature range of 30 to 220 °C with a heating ramp of 10 °C min^−1^ under nitrogen (50 mL min^−1^).

#### 2.5.5 Attenuated Total Reflectance (ATR)

FTIR spectra were obtained using ATR equipped with a Smart Endurance diamond crystal (Spectrum Two ATR, Perkin Elmer, Waltham, MA, United States). Data were collected from 500 to 4000 cm^−1^ over 16 scans in transmittance mode.

#### 2.5.6 Optical Microscopy

The microscopic investigation was conducted using a DM‐L optical microscope (Leica) in transmitted light mode.

#### 2.5.7 Mechanical Strength

The mechanical strength of the four samples was evaluated using the Zwick/Roell dynamometer. The samples were prepared by cutting them to a length of 12 mm and a width of 10 mm, with an effective gauge length of 10 mm for the measurement. During the test, a constant speed of 100 mm min^−1^ was applied to monitor the mechanical behavior of the materials under progressive stress. This approach allowed for the determination of toughness and fracture strain.

### 2.6 Biological Assays

#### 2.6.1 β‐Glucosidase Inhibition Test

β‐Glucosidase inhibition activity was assessed using literature protocol with minor modifications.^[^ ^46^, ^47^ ^]^ Samples (1–10 mg mL^−1^) were mixed with PBS buffer (0.1 M, pH 5) and β‐glucosidase solution (0.005 mg mL^−1^ in PBS). After a 10 min incubation at 37 °C, the reaction was initiated by adding substrate solution (1.5 mg mL^−1^ *p*‐nitrophenyl‐β‐D‐glucopyranoside) and further incubated at 37 °C for 10 min, after which was terminated by adding 700 μL of Na_2_CO_3_ (pH 10). Absorbance was measured at 410 nm. Castanospermine served as the standard inhibitor, and controls included samples without inhibitor (reference) and without enzyme (blank). The percentage activity inhibition was calculated by comparing the absorbance readings of the samples, references, and blanks. All experiments were conducted in triplicate.

#### 2.6.2 Acetylcholinesterase Inhibition Test

The inhibition activity of acetylcholinesterase (AChE) of the extracts was evaluated using a modified Ellman's method.^[^ ^48^ ^]^ In an Eppendorf tube, 160 μL of the extract was mixed with 520 μL of PBS (0.1 M, pH 8.0). To this mixture, 60 μL of AChE solution (0.05 U mL^−1^ in PBS, pH 8.0) and 100 μL of 5,5′‐dithiobis‐(2‐nitrobenzoic acid) (DTNB, 0.25 mg mL^−1^ in PBS, pH 8.0) were added. The reaction mixture was incubated at 37 °C for 30 min. Subsequently, 160 μL of acetylthiocholine iodide substrate solution (0.25 mg mL^−1^ in PBS) was added, followed by another 30 min incubation period at 37 °C. Absorbance was measured at 405 nm. Donepezil was used as the standard inhibitor. Controls included samples without inhibitor (reference) and without enzyme (blank). The percentage activity inhibition was calculated by comparing the absorbance readings of the samples, references, and blanks. All experiments were conducted in triplicate.

#### 2.6.3 Tyrosinase Inhibition Test

Tyrosinase inhibition was assessed following the methodology described by Di Petrillo et al.^[^ ^49^ ^]^ A reaction mixture was prepared by combining 50 μL of the extract with 50 μL of tyrosinase solution (2500 U mL^−1^) and 850 μL of PBS buffer (0.1 M; pH 6.8). This mixture was incubated at 37 °C for 20 min. Subsequently, 50 μL of L‐tyrosine (1.5 mg mL^−1^) was added, and the mixture was incubated again at 37 °C for 20 min. Absorbance was measured at 492 nm. Kojic acid was used as the standard. Controls included samples without inhibitor (reference) and without enzyme (blank). The percentage activity inhibition was calculated by comparing the absorbance readings of the samples, references, and blanks. All experiments were conducted in triplicate.

#### 2.6.4 Antibacterial Activity

The antibacterial activity was evaluated on different strains (*Staphylococcus aureus* ATCC 6538, *Listeria monocytogenes* ATCC 19 112, *Escherichia coli* ATCC 8739, *Salmonella typhimurium* ATCC 14 028, and *Pseudomonas aeruginosa* ATCC 15 442). The test used was the disk diffusion method. Briefly, a fresh suspension of each microorganism (0.5 McFarland) was distributed on Muller Hinton Agar plates. Sterile Whatman paper filters (6.0 mm in diameter) were placed on the plates, and 10 μL of each extract resuspended in water at specific concentration (100 mg mL^−1^ for all compounds except Gallic Acid, which was tested at 10 mg mL^−1^ due to solubility issues in water) were applied to the filters. For each microorganism, a negative control (water) was included. The plates were incubated at the optimal growth temperatures for the different microorganisms (37 °C for *S. aureus*, *L. monocytogenes*, *E. coli*, and *S. typhimurium*; 24 °C for *P. aeruginosa*) for 24 h. After incubation, the inhibition zones (expressed in mm) were measured using a caliper. All experiments were conducted in duplicate.

## 3 Results and Discussion

### 3.1 MASWE Optimization

Several experiments were conducted to determine the optimal conditions for maximizing the recovery of bioactive products from CWW, including low‐weight polyphenols and tannins. CCD was used to determine the optimal conditions for the MASWE step used, followed by an investigation of L/S ratio and confirmation by a kinetic study.

#### 3.1.1 CCD

**Figure 2:**
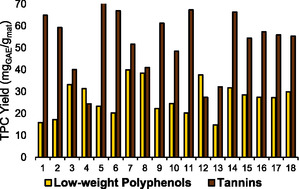
MASWE optimization: tannins and low‐weight polyphenols TPC yield, expressed in mg_GAE_/g_mat_.

Using the set of conditions provided by the CCD, 18 runs were conducted to determine the optimal extraction conditions. As can be noted from Table S3 in Supporting Information, the highest dry yield value (29.04%) was achieved in the run 7, which was conducted at a temperature of 176.0 °C for 14 min at a L/S ratio of 30. Meanwhile, the lowest value (12.51%) was obtained using a temperature of 140 °C and an L/S ratio of 3.2 during an extraction time of 30 min (run 13). However, although the dry yield of the extraction plays a crucial role in providing a prior idea about the process efficiency, the main objective of this work was to optimize the extraction while balancing at the same time the tannins and low‐weight polyphenols. Thus, in **Figure** **2**, the values achieved for the 18 runs in terms of TPC yield are reported. These values were subsequently used for the CCD optimization process. As can be noted from Figure 2, the highest low‐weight polyphenols yield was obtained in run 7 (39.84 mg_GAE_/g_mat_), while the lowest value was related to run 13 (14.74 mg_GAE_/g_mat_). This follows the same observed trend for the results obtained for the dry yield. Focusing our attention on tannins, the outcomes revealed that run 5, conducted at a temperature of 104 °C, with an L/S ratio of 30 and an extraction time of 14 min, led to the highest value (71.08 mg_GAE_/g_mat_). The lowest one belongs to run 4 (24.39 mg_GAE_/g_mat_) which was performed at a temperature of 176 °C and an L/S ratio of 10 for 46 min. To unveil the factors with the most significant impact on the extraction process, Figure S2 in Supporting Information report the Pareto charts obtained for both low‐weight polyphenols and tannin extraction. As can be noted from the figure, in the case of low‐weight polyphenols, a positive correlation exists between temperature and ratio with the output yield.

This trend is in accordance with other findings in the literature showing that high temperature enhances the release of phenolic compounds while a high L/S ratio facilitates mass transfer and reduces the saturation level of the solvent, thus promoting the extraction process.^[^ ^50^, ^51^ ^]^ When focusing on tannins, the Pareto chart reveals that both temperature and extraction time are negatively correlated with tannins’ yield, while the impact of the L/S ratio is positively significant. Other researchers have also reported the decrease in tannins at higher temperature suggesting their thermal degradation.^[^ ^52^ ^]^

**Figure 3:**
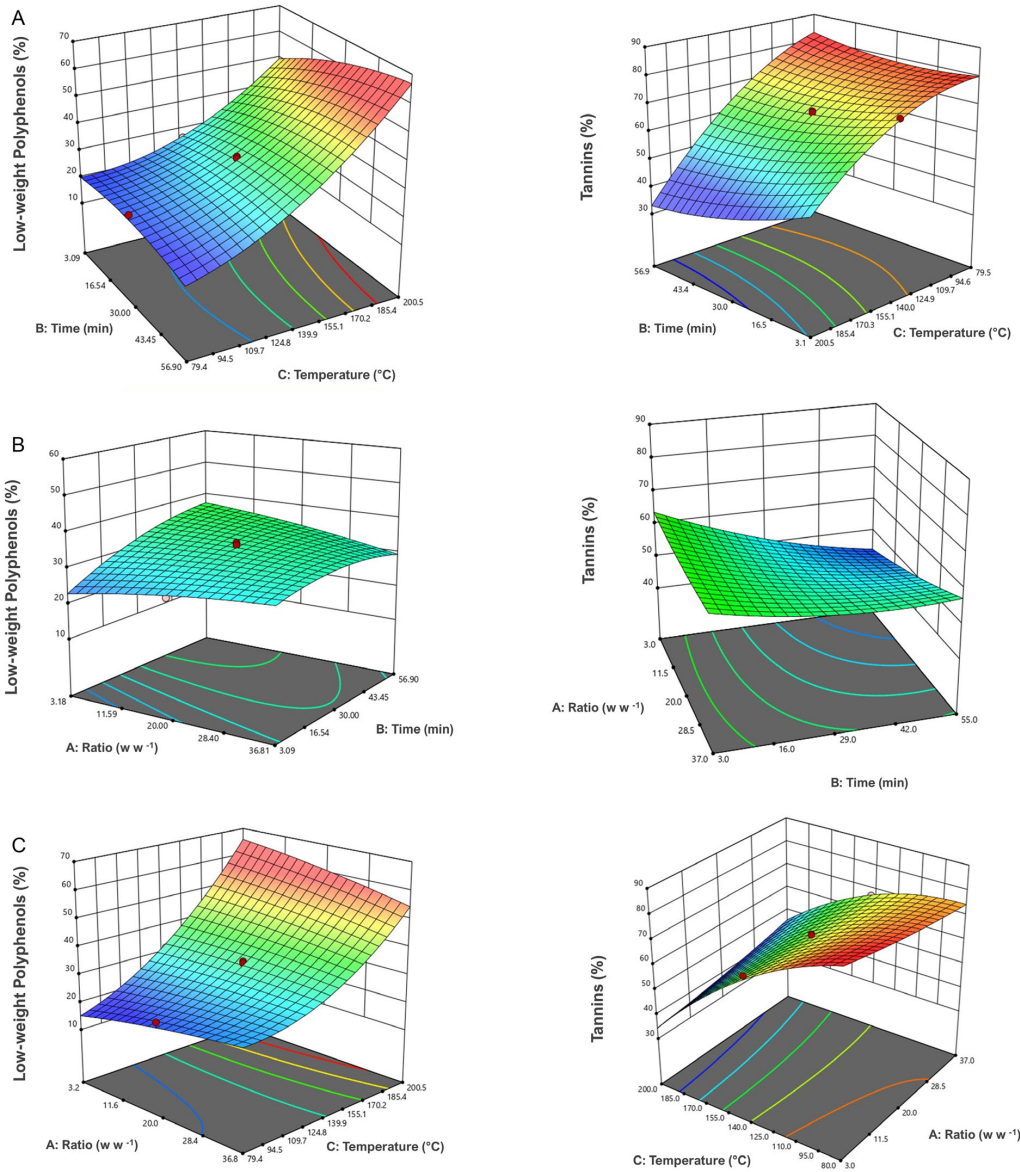
*Effect of process variables on extraction yields of low‐weight polyphenols* and tannins: A) L/S ratio vs. water, B) temperature vs. water, and C) temperature vs. L/S ratio.

To identify the best conditions for achieving the highest yields simultaneously for both low‐weight polyphenols and tannins, the CCD was conducted initially on each of the two classes and then combined. **Figure** **3** shows the effect of the variables used on the yield of low‐weight polyphenols and tannins.

The optimal extraction conditions for each class were as follows. 1) *Low‐weight polyphenols*: the highest T (200 °C. Regr. Coeff. 7.29), the midpoint for the time (30 min, Regr. Coeff. 1.12), and the highest L/S (36.8, Regr. Coeff. 3.41). 2) *Tannins*: the lowest T (79.5 °C, Regr. Coeff. −12.22), the shortest time (3 min, Regr. Coeff. −4.63), and the highest L/S (36.8, Regr. Coeff. 7.33).

As the two optimal conditions differed significantly, a compromise was necessary to optimize the recovery of both species simultaneously, and this was found under the conditions of run 7 (conducted at 176.0 °C, 14 min, L/S 30). However, run 3, which differs only in the L/S ratio, produced similar results. To further investigate the impact of the L/S ratio on the results, a new set of experiments was carried out.

#### 3.1.2 L/S Ratio Investigation

To identify the most appropriate L/S ratio, the new set of experiments was composed by the evaluation of 10 and the intermediate value of 20. This aimed to determine if an L/S ratio of 20 could serve as a suitable compromise, balancing high extraction yield for both compounds while saving water, compared to a ratio of 30.

**Figure 4:**
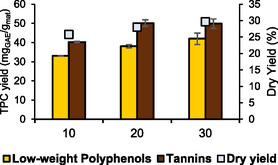
L/S ratio investigation: results expressed in dry yield (%) and low‐weight polyphenols and tannin yield (mg_GAE_/g_mat_).

The results in terms of dry yield (%) and low‐weight polyphenols and tannins yield (expressed as mg_GAE_/g_mat_) are shown in **Figure** **4**.

The intermediate solution of 20 exhibited results in between those observed for 10 and 30 (10: 25.79%; 20: 28.00%; 30: 29.64%). In terms of balancing the two extracted species, the solution at a ratio of 20 showed a slight enhancement of tannins extraction, if compared to others. The polyphenols distribution values, expressed in percentage, are presented in Table S7, Supporting Information. However, the observed differences are negligible, considering also the intrinsic variability associated with the biomass features. Consequently, the ratio of 20 was adopted as optimal due to its contribution to water and energy savings during the process, despite yielding 2% less dry matter than the 30 solution and 2% higher tannins content in the extract.

#### 3.1.3 Extraction Kinetic

**Figure 5:**
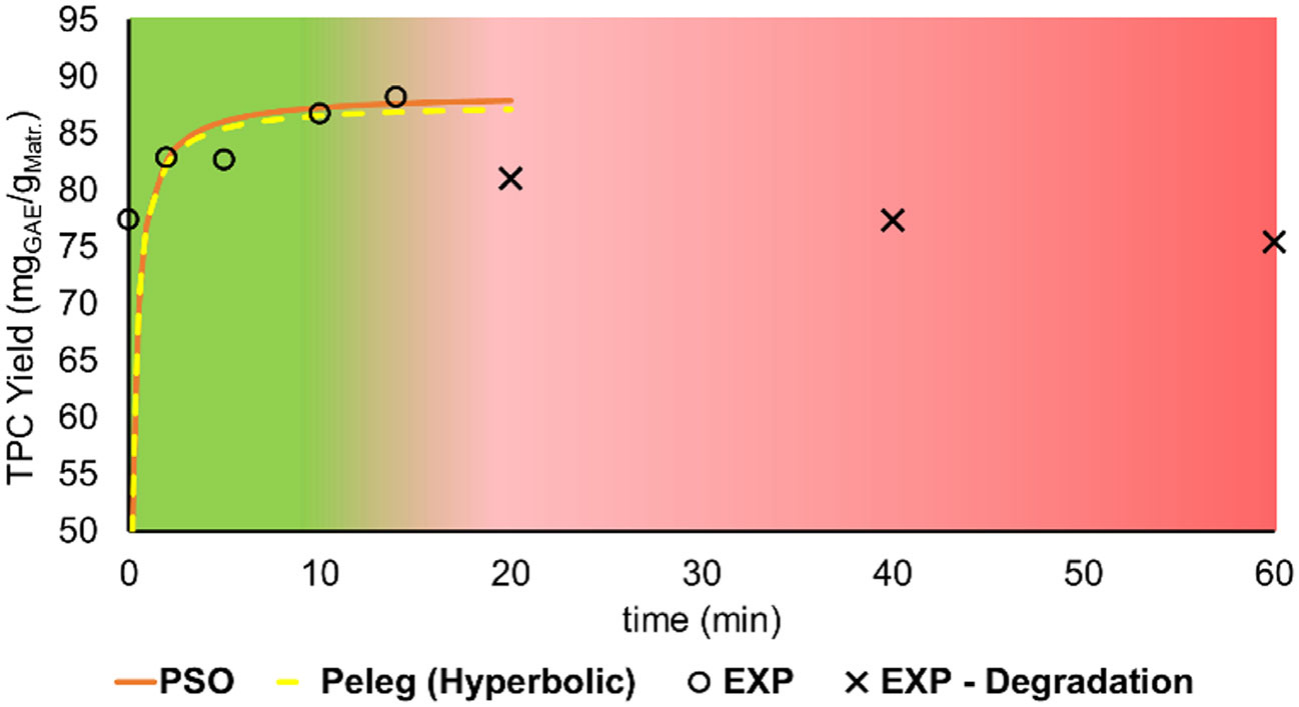
Kinetic evaluation on extraction TPC yield. Experimental data and extrapolated PSO and Peleg models. Degradation onset highlighted with the green/red area.

The extraction protocol optimization was primarily led by the CCD, integrated with the additional study on L/S ratio. Lastly, to better define and understand the extraction trend and mechanism, a time‐resolved investigation was performed by means of kinetic characterization. Firstly, by plotting the evolution of TPC yield in function of process time, it is possible to observe a degradative onset, after 14 min (see **Figure** **5**). For that reason, all the samples achieved above this time were not considered for the kinetic interpretations. The latter was performed evaluating the fitting with different models through linearization approach. PFO, PSO, Peleg, and power law models were applied as “Trend descriptors,” aiming to Q_e_ determination. Conversely, Weber–Morris, Boyd, and Elovich were adopted to shed light on extraction mechanism, relating with, respectively, intraparticle diffusion, liquid film diffusion, and specific chemical interactions.

The best fitting (see **Table** **1**) belongs to PSO model (R^2^: 0.999) with a related Q_e_ of 88.50 (vs. experimental Q_e_: 88.15). In addition, the extraction obeys to both Weber–Morris and Boyd models, demonstrating that either the intraparticle or the liquid diffusion are relevant in the process mechanism (R^2^ of 0.954 and 0.930, respectively). For linear regression, see Figure S3 A–G, Supporting Information.

**Table 1:** Kinetic models fitting and related extrapolated constants.

	Model	R^2^	Constant(s)
Trend descriptors	PFO	–	–
	PSO	0.999	k_PSO_: 0.0798; Q_e_: 88.50
	Peleg (*hyperbolic*)	0.571	k_1_: 0.0015; k_2_: 0.0114 (Q_e_: 87.72)
	Power law	0.787	k_P_: 80.070; n: 0.0335
Mechanism interpretations	Weber–Morris (*intraparticle diffusion*)	0.954	k_ip_: 2.7683; C: 77.726;
	Boyd (*liquid film diffusion*)	0.930	k_f_: 0.229
	Elovich (*specific chemical interactions*)	0.787	α: 4.2265 × 10^12^; β: 0.3506

#### 3.1.4 Tannin Composition

**Figure 6:**
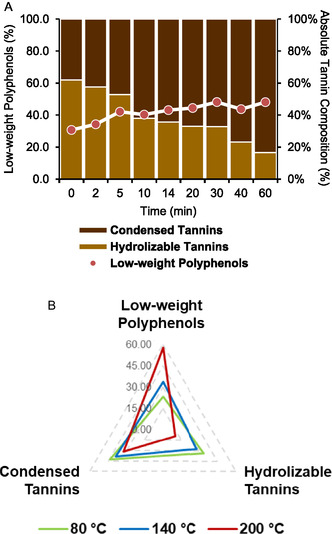
Extract composition (low‐weight polyphenols, HTs, and CTs) according to A) extraction time and B) extraction temperature.

A dedicated study has been performed in parallel with the kinetic, analyzing the effect of time on the composition of tannins in the extract. In Figure S4, Supporting Information, it is possible to observe that this species is the most abundant in the initial stage. However, as the extraction proceeds, the low‐weight polyphenols concentration rises. The two classes of metabolites evolve toward a balance, while the dry yield sees a small decline after 14 min (*approx*. 11.8% of reduction after 60 min). This behavior may suggest that prolonged treatments could lead to the decrease of tannins likely through the hydrolysis of the hydrolyzable fraction. This phenomenon is further supported by **Figure** **6**A,B, which highlights the relation between time and temperature on the composition of the two different classes of tannins in the extract (condensed and hydrolyzable). The 100% stacked columns clearly point out how, after the first 5 min, the composition turn towards condensed structures, while the gallo‐ and ellagitannins decrease throughout all the process, in favor of low‐weight polyphenols. An additional comment can be made in relation with the poor fitting with Elovic's model (see Table 1), peculiar for chemical Interactions kinetics. Thus, it is possible to hypothesize that the hydrolysis phenomena are not directly involved in the extraction but are likely to happen once the metabolites are freed in the liquid media.

For what concerns the temperature‐related investigation, the extremes of the CCD have been selected, together with an intermediate value (80, 140, and 200 °C), fixing the average process time of 30 min. In Figure 6B, it is possible to observe a general contraction of tannins as the temperature increase, in particular for hydrolyzable ones at 200 °C (from 33.71% at 80 °C to 9.75% at 200 °C). This trend is concurrent with an increase in low‐weight polyphenols, as similarly seen for time‐resolved studies. Hence, it could be likely related with the release of phenols once belonging to tannins, in connection with high temperatures. In conclusion, as observed from the kinetic study, together with the tannin trend, the time suggested from CCD (14 min) can be considered optimal as it maximizes TPC yield and achieve polyphenols distribution close to 50% of each species. While a better polyphenols balancing was achieved with a 60 min extraction, the increased process time do not motivate the overall energy consumption (and thus the reduced sustainability) of the process compared to the negligible product enhancement.

Consequently, optimal conditions for the MASWE of CWW can be defined as 176 °C, L/S 20, and 14 min.

### 3.2 Membrane Filtrations

**Figure 7:**
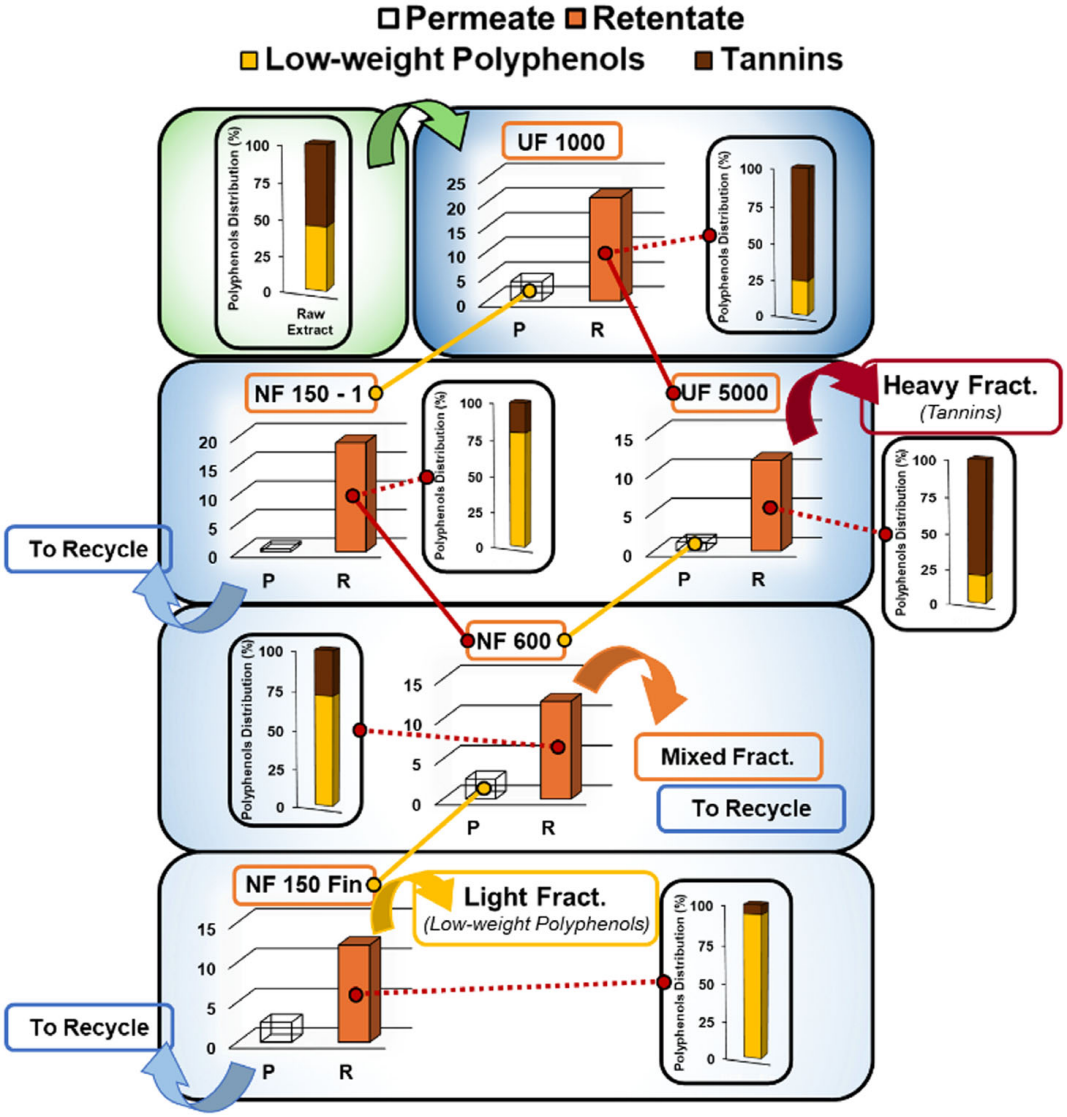
Extract concentration (mg mL^−1^) and polyphenols distributions (%) in membrane fractions.

A membrane filtration protocol using a cascade approach was applied to the CWW extract to fractionate and concentrate the components. The main aim of this procedure was to isolate a fraction enriched in tannins. Further filtration steps were then applied to maximize the water recycle and the metabolite valorization, recovering the light part of the product, primarily composed of low‐weight polyphenols. To follow the membrane filtration trend, different parameters have been screened, namely *i*) extract concentration (mg mL^−1^), *ii*) low‐weight polyphenols/tannin distribution (%), *iii*) TPC selectivity (mg_GAE_/g_ext_), and *iv*) TSC (mg_Glu_/g_ext_). Those values are reported in **Figure** **7** and S5, Supporting Information. The starting raw extract was characterized by 13.86 mg mL^−1^ concentration, an overall tannin content of 54.84%, a general TPC selectivity of 322.11 mg_GAE_/g_ext_ and a TSC of 358.54 mg_Glu_/g_ext_.

The general feature of membrane filtrations allows us to discriminate, for every step, a concentrated fraction (retentate) and a strongly diluted one (permeate). Thus, in a cascade approach, we expect to concentrate bioactives in the retentates while recovering great amount of water in permeates with very low concentrations of nonactive species (i.e., salts and sugars) that are eligible for recycling. Figure 7 can help to visualize the flow distribution throughout the process. Starting from this concept, tannin have been accumulated in retentates by different membrane cut‐off, from 54.84% of the raw extract to 76.97% of RUF_1000_ and to 80.10% of RUF_5000_, identified as the final tannin‐rich fraction (HF). Consequently, molecules with a molecular weight between 1000 and 5000 Da were collected in permeates (PUF_1000_ and PUF_5000_), which have been sequentially concentrated by nanofiltration (NF_150_ and NF_600_, respectively). PUF_1000_ was processed in NF_150_‐1, and the retentate was characterized mainly by low‐weight polyphenols (RNF_150_‐1 tannins: 20.09%). The resulting PNF_150_‐1 recovered the 89.1% of water and, due to the low concentration (0.54 mg mL^−1^), was destined to recycle as discussed in the **Water Recycle section**. To minimize losses and maximize water reuse PUF_5000_ was exploited to dilute and diafiltrate RNF_150_‐1, processing the combined mixture through NF_600_. The retentate, even if primarily composed by low‐weight polyphenols, saw a general concentration of residual tannins from PUF_5000_ and RNF_150_, (28.34%). This fraction has been identified as “Mixed Fraction” (RNF_600_, MF) and reintegrated at the beginning of the process to fractionate it again with a loop approach, as discussed in the **Water Recycle section**. The resulting PNF_600_ underwent further concentration (NF_150_ Fin), recovering 82.7% of water and resulting in a final stream of concentrated low‐weight polyphenols (tannins: 5.89%), identified as LF (RNF_150_ Fin). This latter result contributes to support the fractionation effectiveness of different classes of compounds by means of membrane. The filtration protocol allowed also to separate the HF (and LF) from nonactive compounds such as salts and saccharides (naturally contained in the raw extract). This can be verified by the enhancement of the TPC selectivity (see Figure S5, Supporting Information) in both RUF_1000_ and RUF_5000_. On the contrary, this parameter strongly decreases in permeates.

**Figure 8:**
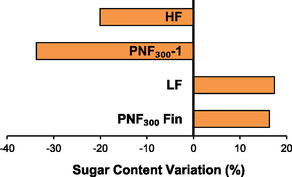
Variation in sugar content (%). Reference value set as “0” is the raw extract, starting material of the membrane filtration.

As mentioned, the main fractions were characterized also in terms of sugar content (see Figure S5, Supporting Information). Adopting the raw extract as a reference (358.54 mg_Glu_/g_ext_), **Figure** **8** highlights that HF results in a considerable sugar content reduction (−20%), which is even surpassed by PNF_150_‐1(−33%). As expected, those sugars are conveyed towards the final stages of the filtration process, where LF and the final PNF_150_Fin reach a +17% and +16%, respectively.

The choice to adopt the sequence “1000 Da‐5000 Da” for the UF is supported by different practical considerations. Applying a higher MWCO from the beginning (i.e., 5000 Da) would have given a less pure tannin fraction, an effect amplified by the fact that MWCOs are based on hydrodynamic volume, which does not account for the bulky and complex structures of tannins. Small polyphenols, under such conditions, may also struggle to pass through the membrane, leading to a less efficient separation. To avoid that, a prefractionation at 1000 Da was conducted, followed by 5000 Da refining, in order to enhance the tannin purity getting rid of all the components of mass comprised between 1000 Da and 5000 Da, once the matrix effect had been reduced due to the prefractionation. The sequential use of membranes generally has the desired side‐effect to reduce the fouling, simplifying the filtration process. The whole procedure accounted for an overall raw extract mass fractionation of 74.18%, distributed as 24.66% HF, 24.05% MF (which can be subsequently reintegrated into the process), and 10.28% LF (leading to a 1.92% for the Res. Pur.). Residual amounts are contained in the permeates (≈15.19%). Those values are in agreement with the considerable population of high molecular weight in the achieved extract as well as with the removal of salts and sugars from LF to Res. Pur. In particular, the latter enhance the TPC selectivity recovering up to 80% of the LF polyphenols, thus contributing to the full valorization of bioactives from CW and effectively closing the loop of the process.

#### 3.2.1 Water Recycle

**Figure 9:**
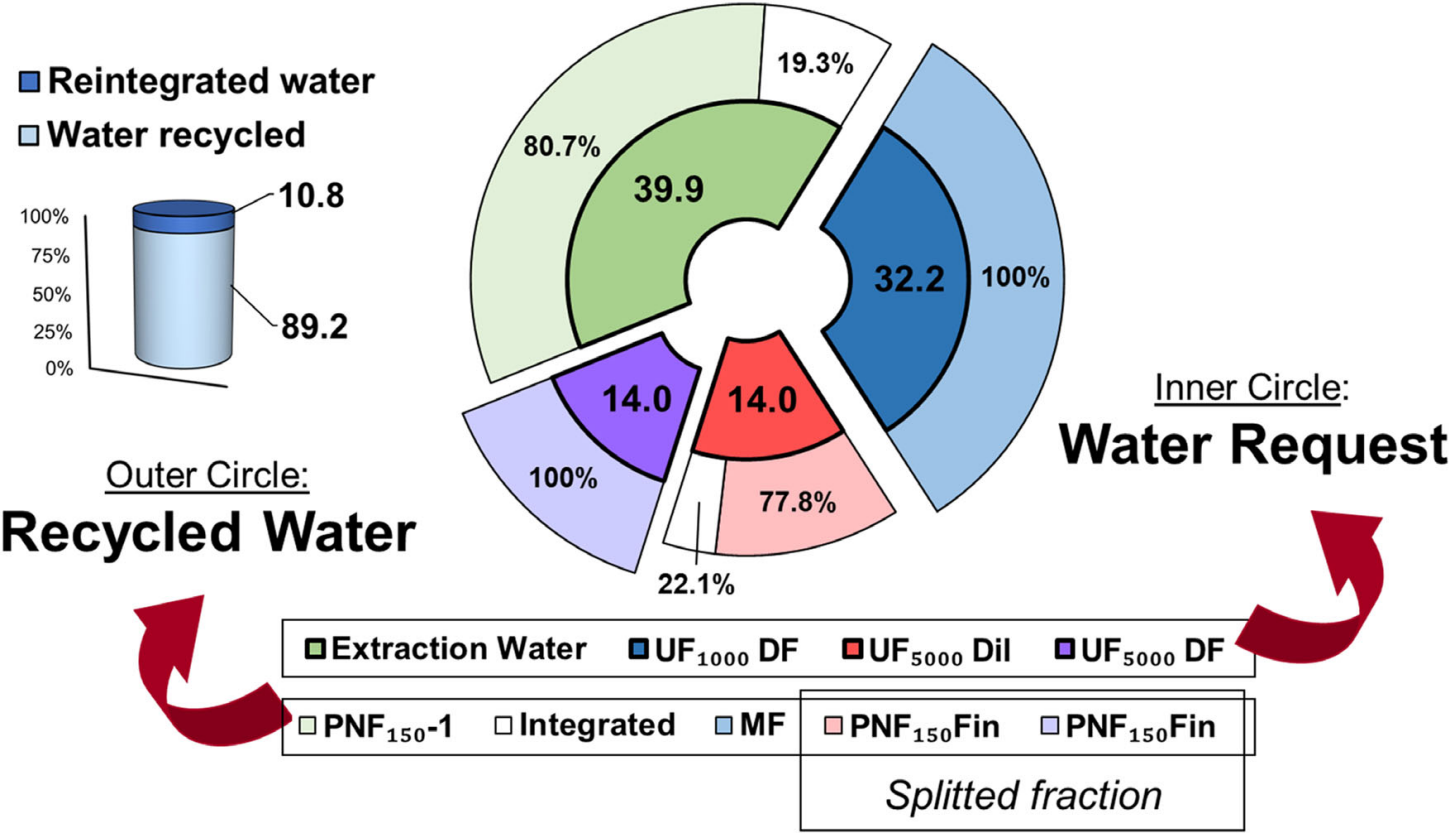
Water recycle for CWW valorization. Inner circle: water required from the process (extraction, diafiltrations, and dilutions); outer circle: aqueous by‐product reuse distribution added with required water integration.

The water request for the CWW valorization protocol reach a comprehensive ratio of 1:50, in respect to the initial biomass. This amount can be divided into extraction (39.9%), diafiltration (32.3% plus 14.0%), and dilution (14.0%) (**Figure** **9**, inner circle). The fractionation protocol allows to recover concentrated bioactives streams splitting them from few strongly diluted sources of water. Those latter can be recycled inside the membrane filtration itself, providing a water saving of ≈89%, with the positive side‐effect of reintroducing in the process the minimal amount of actives lost through the membrane cut‐off. In such a way, every fractionation cycle could take charge of the by‐product produced by the previous. This feature is particularly interesting for the MF, still containing tannins and with a non‐negligible concentration. The reintroduction of this fraction as diafiltration liquid onto the UF_1000_ leads to reintegrate polyphenols in the process as well, together with water. Figure 9 shows how it is possible to fulfil the water request with the different streams, together with the aliquot that need to be reintegrated due to solvent evaporation.

This shortfall, accounting approx. for 10% of the total amount, is due to the drying process of HF and LF. Further studies could involve the aqueous fraction, residual after the resin purification of the low‐weight polyphenols.

### 3.3 HF—Application

#### 3.3.1 Biopolymers

Combining PVA with other bioderived and renewable substances to improve properties of biofilm is a new challenge to obtain functional biopolymers. The chemical structure of PVA, rich in —OH groups, allows the possibility of interaction with other compounds. In this case, we investigated the combination of PVA with tannins and pectin derived from biomass. To obtain good mechanical characteristics, the addition of a crosslinker was necessary.^[^ ^53^ ^]^ Glutaraldehyde is commonly used for this type of applications;^[^ ^44^, ^54^, ^55^ ^]^ however, it is known that the synthesis and production of this petrol‐based chemical are environmentally impactful due to the use of heavy catalysts and complex chemical reactions,^[^ ^56^ ^]^ and its toxicity poses significant risks to aquatic organisms.^[^ ^57^, ^58^ ^]^

In this study, ItA was used, as a more sustainable and bioderivable option, being a derivative from itaconic acid, a platform chemical of great interest, derivable from biomasses.^[^ ^59^, ^60^, ^61^ ^]^ Previous studies established positive effects with the addition of ItA in resin biocomposites, especially improving thermo‐mechanical properties.^[^ ^62^ ^]^ Gly, as commonly reported in the literature, was added as a plasticizer to obtain a strong, durable, and elastic polymer.^[^ ^63^, ^64^ ^]^ According to the literature, it is most likely that ItA interacts with PVA through esterification. Similarly, the —OH can be esterified or they can interact with to PVA with hydrogen bond.^[^ ^65^ ^]^ Gly is expected to act as a plasticizer, facilitating the formation of a well‐defined reticular structure through hydrogen bond formation between the components. Those conclusions well fit the aforementioned literature with experimental data reported in following characterizations (see the **Biopolymer Tests**). The evaluation of pectin as additive is primarily due to their recognized role as valuable bioderived material for polymers compounding, together with its large availability as “bulk chemical” and for its aptitude to be tailored modifying the esterification degree (and chain length). By using a biobased polymer extracted from waste materials, we aimed to enhance the sustainability of the preparation, decreasing the percentage of PVA used.

The first tests performed showed rigid and fragile systems, not able to pass through the drying phase without breaking after the volume contraction, or in alternative, after minimal manipulations. Thus, the first evaluation regarded simply the wholeness of the material produced at the end of the protocol, including surface uniformity. The screened parameters were the quantity of HF (35, 40%, and 45%), the amount of Gly (0, 10, and 20%), the presence of ItA, and the ratio between PVA and PF (0:100, 25:75, 50:50, and 75:25). All the conducted tests are displayed in Table S8, Supporting Information; it is possible to notice that the amount of HF had to be balanced to have uniformity and to avoid having aggregates isolated from the rest of the matrix meanwhile having good mechanical characteristic: 40% appeared as the best compromise. The presence of Gly, in the quantity of 20%, was necessary to give to the polymer a good elasticity and therefore avoid the breaking of the sample. The IA was fundamental to facilitate the creation of the polymer bonds, and without it, the polymer had not good mechanical behavior. The blending with PF was interesting both for the aspect uniform and opaque and for mechanical strength, in the case of sample 9, with the lowest amount of PF. An excess of pectin, on the other hand, produced a structure too rigid to withstand physical solicitations.

To better assess the characteristics of the prepared biopolymers, specific analyses were conducted on selected samples. Reference 1 and reference 2 served as blanks to better understand the influence of ItA and Gly. Thus, the following results of further analyses attest to intrinsic characteristic of the biopolymer and mechanical behavior.

## 4 Biopolymers Characterization

To evaluate the mechanical and thermal characteristics of prepared biopolymers, several tests were conducted (TGA, DSC, optical microscopy, and mechanical strength test) on the selected polymers (samples 4 and 9, Reference 1 and Reference 2) and on the starting materials (PVA and ItA).

### 4.1 TGA

Figure S6, Supporting Information, shows the different weight loss behavior in function of temperature for the biopolymer sample 4 and 9 together with the Reference 1 (without ItA and Gly) and Reference 2 (without Gly). Reference 1 and reference 2 showed similar behavioral, with an initial weight loss of ≈3%, between 30 and 140 °C, probably related to residual humidity evaporation. However, the differences are shown at higher temperature: sample 12 shows a main degradation which end at 460 °C, and Reference 2 presented an additional weight loss phase between 365 and 460 °C.

The final residue at 750 °C is 17% for reference 1% and 19% for Reference 2, pointing out a thermal residual stability slightly higher for the latter. Samples 4 and 9, containing Gly, presented a different behavior. Both are not showing significant initial weight loss, suggesting the contribution of glycerol in reducing the retained humidity. Sample 4 degrades in two main phases: first weight loss between 150 and 230 °C, probably linked to the initial decomposition of glycerol and less stable components, and a second one between 430 e 470 °C, with a final residue of 10.5%. Sample 9 showed a more prolonged initial loss, extended between 150 and 360 °C, followed by a second phase between 360 and 470 °C, remaining the highest residue between all the samples, amounting to 27% at 750 °C. This high residual weight indicates that the combination of PVA, tannins, and pectins led to the formation of more thermally stable reticulated network. The differences between the samples clearly highlight how the presence of Gly and the different composition influence the thermal behavior: the samples with Gly (4 and 9) show a higher initial stability and a more complex degradation pattern, while the samples without (Reference 1 and Reference 2) distinguish for a linear degradation and more substantial residues.

### 4.2 DSC

To better investigate the behaviors of the chemical species involved, DSC analysis was performed (Figure S7, Supporting Information), including also on pure ItA and PVA. The DSC thermogram of pure ItA shows a melting peak at 67 °C and a self‐crosslinking peak at 214 °C, with a similar peak observed in sample 4 and Reference 1 containing PVA and tannins. In Reference 1, which does not contain ItA, the formation of crosslinked bonds can be attributed to the presence of hydroxyl groups in both PVA and tannins, which may participate in condensation reactions. This suggests that even in the lack of a specific crosslinking agent like ItA, the material can still develop some kind of network when heated up. In sample 4, the peak shift at ≈203 °C respect ItA alone suggests interactions between ItA's functional groups and the hydroxyl groups in PVA and tannins. The presence of a less pronounced peak suggests that the crosslinking process is largely complete, leaving few residual functional groups available for further reactions. In sample 9, where PVA is partially replaced by pectins, no significant peaks are observed in the same temperature range. This result could indicate that the presence of pectin led to a more complete and effective crosslinking process. In the analysis of pure PVA, a peak is observed around 225 °C, which can be attributed to an intramolecular crosslinking phenomenon of PVA with itself. This peak highlights the distinctive thermal behavior of PVA under high temperatures, demonstrating its ability to form chemical bonds independently. The collected data support the interactions between the species present in the mixture while forming the polymer (PVA, ItA, tannin‐rich fraction, and Gly).

### 4.3 Optical Microscopy

In Figure S8, Supporting Information, the images captured with microscope are shown, for the four samples. Reference 1 presents a relatively uniform distribution, showing some irregularities in the form of bubbles or scattered inclusions. These characteristics are related to the matrix composition, based on PVA and tannins, without any additives, as Gly, or crosslinker as ItA. Darker regions are also observed, which could represent local tannins aggregates, as they tend to form separate microphases in the absence of compatibilizing agents. The presence of these irregularities could negatively affect the stability and the mechanical properties of material. In contrast, sample 4 shows a more defined and uniform structure, with respect to Reference 1. The granular area observed could represent Gly. Reference 2 presents a surface characterized by lines and striations distributed on the observed area. The darker streaks suggest the presence of microfractures or structural inhomogeneity, probably related to the absence of Gly, which reduce the flexibility of the material, making it more fragile during processing. Moreover, the presence of ItA, promoting the crosslinking process, contributes to a higher stabilization of PVA chain and tannins, significantly reducing the mobility, conferring a more rigid and brittle structure to the material. Finally, sample 9 displays an overall homogeneous structure, characterized by a regular granular distribution. This suggests that the presence of pectins may have promoted good compatibility between the components.

### 4.4 ATR

The ATR spectra of sample 4 and sample 9 have been collected and compared with the single components (HF, pectin, and blank as a reference). As expected, the diagnostic peaks of PVA and pectin (Figure S9, Supporting Information) are predominant in the samples, together with characteristic signals belonging to both aliphatic and aromatic structures of HF. It worth notice that several band shifts are visible, pointing out an effective interaction between the different components of the biopolymer, in agreement with the thermogravimetric analysis. Detailed results and peak assignation have been reported in Figure S9–S11, Supporting Information and related discussion.

### 4.5 Mechanical Strength

Reference 1 and Reference 2 showed significantly lower mechanical resistance, breaking immediately under stress. This behavior can be attributed to the absence of Gly, which in samples 4 and 9 acts as a plasticizer, improving flexibility and force distribution within the material as expected.^[^ ^62^, ^63^ ^]^ For sample 4, the break force is 213 cN, with an elongation at break of 12.4% and a toughness of 338 MPa. These results reflect a moderately strong material but relatively rigid. Sample 9, in contrast, with a break force of 1280 cN, an elongation at break of 29.6%, and a toughness of 20 031 MPa, stands out significantly for its mechanical strength and ability to deform before breaking. The combination of PVA, tannins, and pectins has likely contributed to improving force absorption, distributing mechanical stress more evenly. This has resulted in a material with high strength and flexibility, making it ideal for applications requiring good mechanical stability.

### 4.6 TPC

After the characterization of the polymers, sample 9 was identified as the best performing for all the investigating characteristics. TPC and antioxidant activity of this polymer were investigated. Regarding the TPC analysis, the identified value was 95.33 mg_GAE_/g_Polymer_, demonstrating that after the dissolution in water the polymer could release such that quantities of polyphenols that can be redetected with Folin–Ciocalteu test.

### 4.7 Antioxidant Activity

Both the surface and the release activity are investigated in term of antioxidant features, varying the contact with the radical probe (direct or indirect, respectively). The surface activity was determined in a TEAC of 0.35 mmol_Trolox_/g_Polymer_ while the release one in 0.31 mmol_Trolox_/g_Polymer_. These results suggest that most of the active species are released by the material during the test (conduced in MeOH). On the contrary, a lower value for the release test would have indicated that mainly metabolites fixed on the polymer surface were responsible of the DPPH· scavenging. Further tests have to be conducted investigating different type of solvents, to exclude the release of species during the assays, thus specifically evaluating the surface activity feature.

Considering the antioxidant features of the polymer in respect to the HF loading (25.81% w/w), the polymer maintains 8.66% and 7.87% of the fraction's original performance (see the paragraph **Antioxidant activity**), according to the surface activity and the release one, respectively. The collected data suggest that the HF active fraction exhibits reduced availability probably due to its involvement in the polymer backbone, resulting also in its partial entrapment within the polymeric network. This confinement limits its accessibility and reactivity, thereby decreasing its effective antioxidant features. Nonetheless, the polymer retains significant antioxidant capacity, which can be exploited for potential applications. Further studies are needed to evaluate the potential release of the active compound over time. This behavior could be interpreted as a type of encapsulation, which may contribute to extended shelf‐life and prolonged maintenance of antioxidant activity, by gradually releasing the active compound and thereby extending its effect over time.

### 4.8 Soil Burial Test

**Figure 10:**
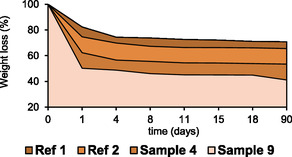
Evaluation of weight loss (expressed in %) of polymer during burial test.

The biodegradability of the prepared polymers was evaluated through burial test, and it is presented in **Figure** **10**, expressed as weight loss with respect to the starting point (%). Each sample exhibited significant weight loss during the first day (for sample appearance see Figure S12, Supporting Information).

Following this initial phase, the weight reduction became more gradual, eventually reaching an apparent steady state. At the end of the evaluated period, the residual polymer was substantial, indicating that a significant portion was still present, in different proportions, according to the type of polymer. The two reference samples appear to be the most affected (70.8 and 65.5% of weight loss, respectively). Samples 4 and 9 on the contrary showed the lower mass weight over the 90 days period (53.5% and 41%, respectively). It is possible to conclude that, in general, the crosslink provided by ItA enhances as expected the stability of the material as well as HF and pectin addition. In general, the fact that samples 4 and 9 are more stable than the references is an encouraging result because it suggests that tannin fraction stabilize the PVA framework, extending its range of applications. In fact, a certain water resistance should be provided for active packaging or mulching films, possibly by means of biodegradable and bioactive components. Thus, the wetting step of the test possesses a crucial role in the biopolymer destruction, since a direct immersion lead into a complete dissolution of all the samples in 3 days. Further evaluations could investigate different water additions.

## 5 LF—Application

### 5.1 Resins Purification

**Figure 11:**
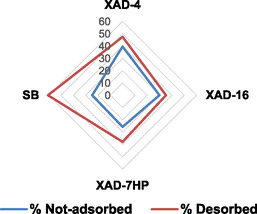
Screening of different resins for LF purification.

The LF (RNF_150_) results as a final by‐product from the tannins enrichment protocol (HF) and contains an important amount of sugars and salts. To enhance the quality of this fraction, elevating a potential “residue” to a side‐product is necessary to get rid of those nonbioactive compounds and enhance the selectivity in polyphenolic components. This task can be affectively achieved by means of resins purification. To investigate the most suitable system, a screening of four different resins (XAD‐4, XAD‐7 HP, XAD‐16, and SB) has been conducted. The evaluation was based on the ratio between the efficiency of adsorption and desorption. This approach allows to determine the stationary phase with a good affinity with the polyphenols mix but weak enough to guarantee the most quantitative recovery and low losses. From an experimental point of view, more accuracy has been achieved on nonadsorbed TPC evaluation. followed by desorption determination. To normalize the results, they are expressed as percentage in respect of the mother solutions (see **Figure** **11**).

The SB resin showed the best results, both in terms of adsorption and desorption step. XAD‐7 HP seemed to have a similar behavior with respect to SB in the adsorption step, but in the desorption step, it was less effective (37.85% vs. 60.49%). SB was considered the optimal type of resin to be used for this purpose.

### 5.2 Resin Purification Optimization—CCD

#### 5.2.1 Adsorption

**Figure 12:**
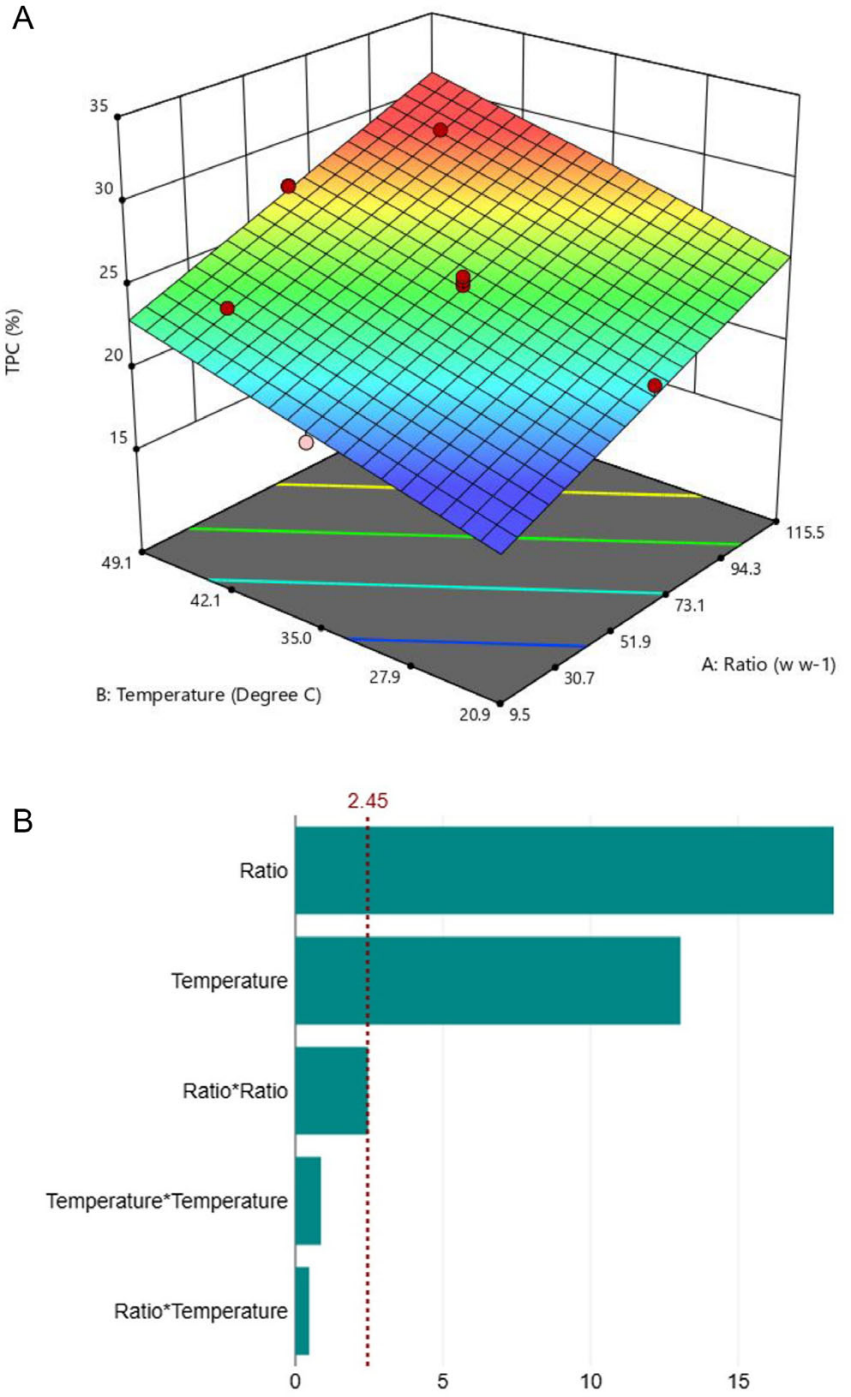
Adsorption process CCD optimization. A) Effect of parameters on the TPC and B) Pareto chart for parameter relevance (*p* = 0.1).

Once the most promising stationary phase was determined, a fine tuning of the purification protocol was conducted with the help of CCD. The first step was devoted to maximize the adsorption of LF polyphenols, with 12 different tests. The obtained values were normalized on feed solution and expressed in percentage. The results are displayed in Table S4, Supporting Information. The highest value was obtained in the nonadsorbed fraction of run 4 (29.20%) conducted using a temperature of 100 °C and a ratio of 45 mg_ext_/g_resin_, while the lowest value was found in the fraction linked to run 1 (18.85%) performed at 25 °C and with a ratio of 25 mg_ext_/g_resin_. No significant difference was found between the later experimental value and the predicted content using the CCD (18.98%) confirming the accuracy of the model. The effect of the adopted parameters on TPC during the adsorption process is illustrated in **Figure** **12**.

As illustrated in Figure 12A,B, both temperature and weight of extract to gram of resin ratio exhibit a high impact on the adsorption process. Medium temperature and relatively low ratios were associated with low adsorption of polyphenols. In particular, high temperatures have already been reported to minimize the process efficiency since the adsorption is known to be exothermic.^[^ ^66^, ^67^ ^]^ Thus, conditions of run 1 (25 °C, and a 25 mg_ext_/g_resin_ ratio) were therefore fixed with the objective of optimizing the desorption process.

#### 5.2.2 Desorption

**Figure 13:**
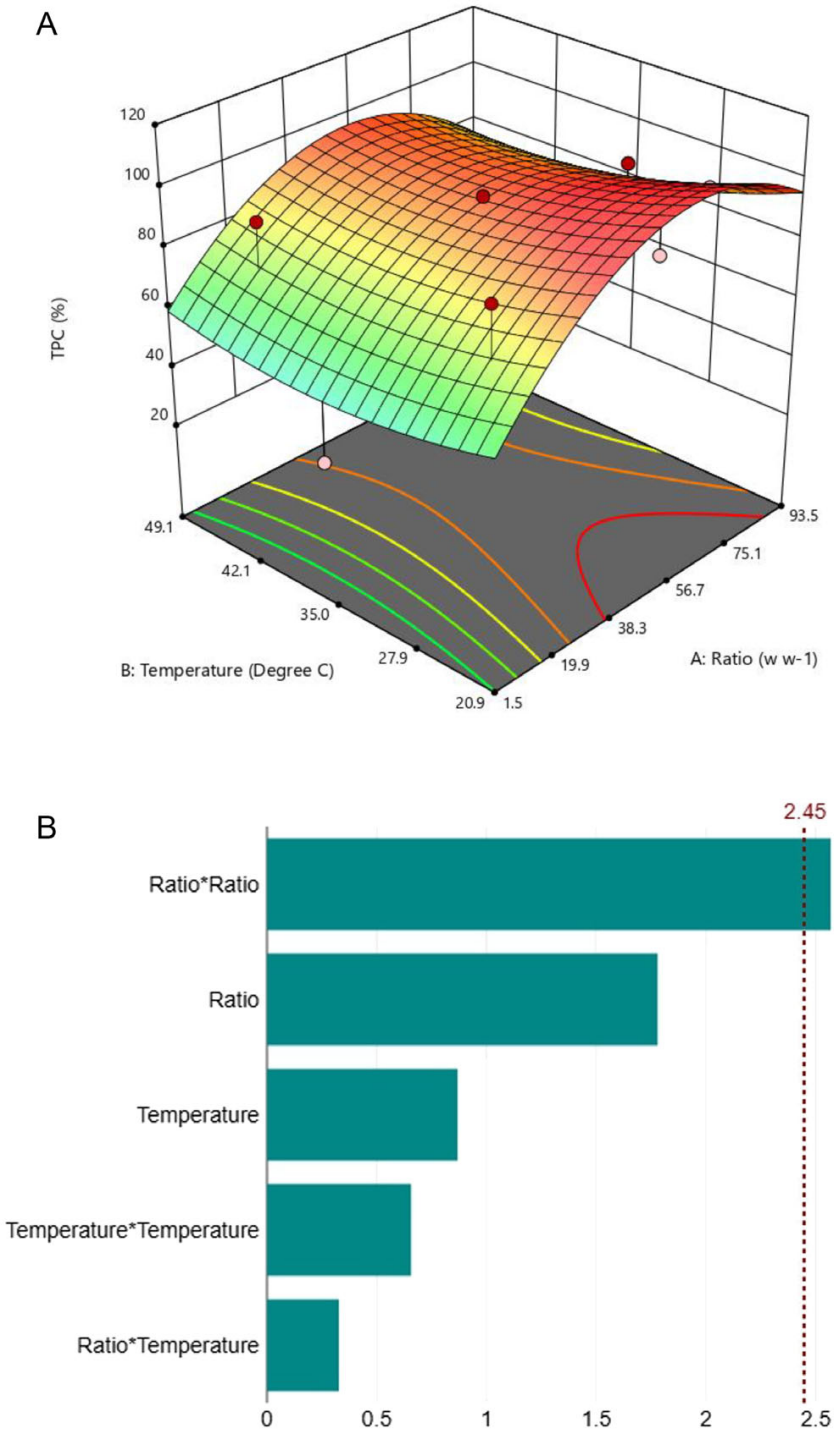
Desorption process CCD optimization. A) Effect of parameters on the TPC and B) Pareto chart for parameter relevance (*p* = 0.1).

After the determination of the most promising conditions for the adsorption step, the following step was dedicated for investigating and optimizing the desorption step, to recover all adsorbed polyphenols and reduce losses. With 12 differen*t* tests, identified based on the CCD, as previously mentioned, the effect of both temperature and the ratio were investigated. Results were normalized on the real adsorbed values and expressed as percentages and displayed in Table S5, Supporting Information. The highest desorbed content of polyphenols (99%) was found in run 12 linked to a temperature of 20.86 °C and a ratio of 47.5 (mL_solvent_/g_resin_). No statistical difference has been found between the experimental value (99%) and the predicted one (102.24%) at a significance level of 5%. The impact of the adopted parameters on the TPC during the desorption process is represented in **Figure** **13**.

The solvent volume to resin ratio has been identified as the most significant factor during the desorption process (Figure 13B). The optimal value, being approximately in the middle of the investigated range, represents a suitable compromise between effective desorption and subsequent product isolation. This balance ensures efficient performance while minimizing solvent evaporation costs, thus preventing excessive waste. The use of low temperature and a relatively modest amount of solvent leads to saving energy end reducing process costs, a significant factor considering also that this protocol is applied on a by‐product fraction from the main process.

Considering the aforementioned findings, it is noteworthy that the optimal parameters for the adsorption and desorption processes were determined to be as follows: *i*) adsorption: 25 °C and 25 mg_ext_/g_resin_ and *ii*) desorption: 20.86 °C and 47.5 mL_solvent_/g_resin_.

#### 5.2.3 Kinetic Study

An adsorption process typically involves up to four distinct yet interconnected stages. These include the following: the transfer of the adsorbate from the solution to the external surface of the adsorbent, the interaction of the adsorbate molecules with active sites on the adsorbent's surface, the migration of the adsorbate from the surface into the intraparticle active sites within the adsorbent matrix, and the interaction of solute molecules with the internal surface active sites or material relaxation within the adsorbent.^[^ ^38^ ^]^ The overall rate of adsorption is governed by one or more of these stages, depending on the physicochemical properties of both the adsorbent and the adsorbate. These stages may occur either concurrently or sequentially.

**Figure 14:**
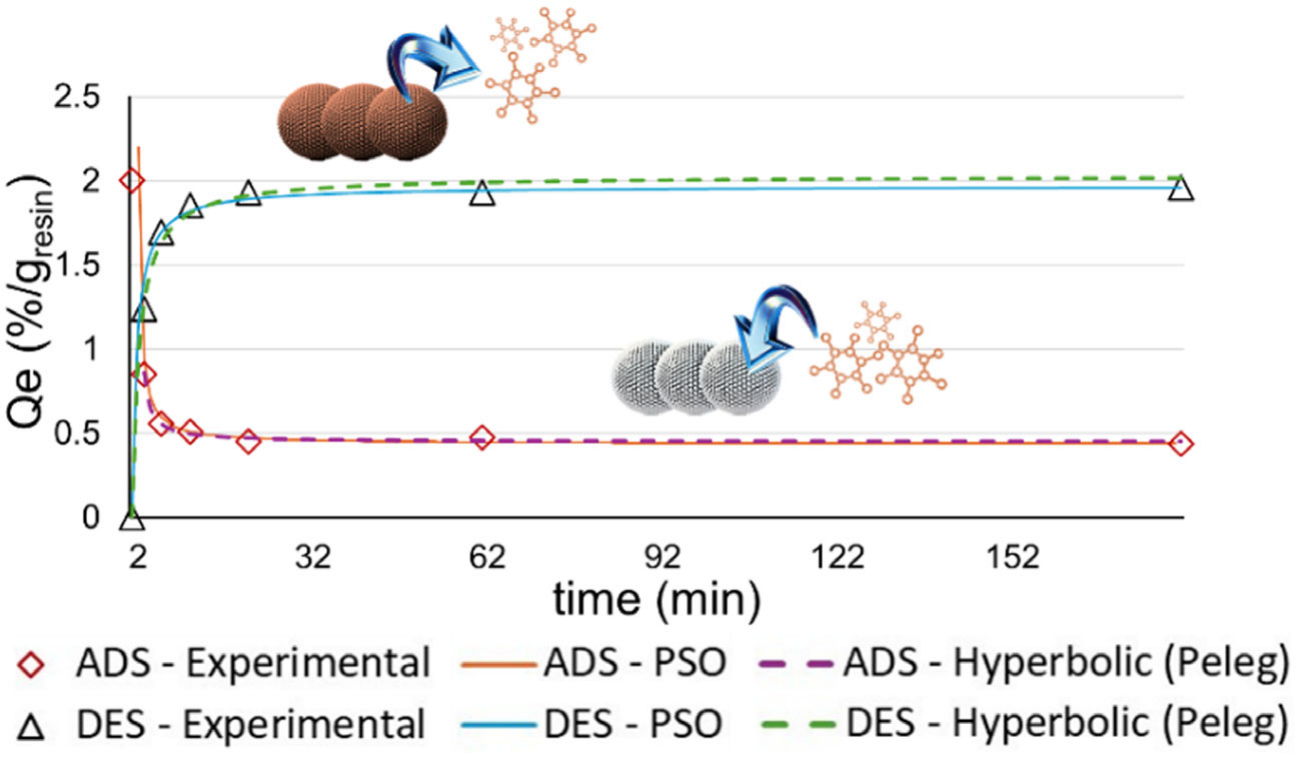
Kinetic study of polyphenols adsorption/desorption on SB. Experimental data and PSO and Peleg model fitting.

To identify the rate‐determining step(s) among the four stages, the adsorption of polyphenols onto SB, as well as the desorption, was investigated using different kinetic models. Related fitting quality are reported in **Table** **2**. The linearization plots can be found in Supporting Information (Figure S13 A–F and S14‐A‐G, Supporting Information). Both adsorption and desorption appear to obey to the PSO kinetic, with a good fitting also for Peleg model. R^2^ for all the other models are very low. **Figure** **14** reports the two PSO and Peleg model curves, together with the experimental values, showing good agreement between experimental and calculated data.

**Table 2:** Kinetic models fitting and related extrapolated constants.

		R^2^	
	Model	ADS	DES
Trend descriptors	PFO	–	–
	PSO	0.999	0.999
	Peleg (*hyperbolic*)	0.973	0.980
	Power law	0.682	0.632
Mechanism interpretations	Weber–Morris *(intraparticle diffusion*)	0.368 (0.968–0.509)	0.395 (0.908–0.914)
	Boyd (*liquid film diffusion*)	0.179 (0.878–0.479)	0.685 (0.968–0.958)
	Elovich (*specific chemical interactions*)	0.626	0.669

The PFO kinetic model posits that the adsorption rate is primarily governed by the diffusion of the adsorbate molecules across the surface of the adsorbent. In contrast, the PSO kinetic model is more applicable to the adsorption processes that requires extended durations to achieve equilibrium, particularly when the adsorption rate is significantly influenced by the chemical interactions between the adsorbent's active sites and the adsorbate throughout the adsorption process.

These models provide critical insights into the mechanisms and time dependencies of adsorption under varying conditions.^[^ ^68^ ^]^ This interpretation fit with the structure of SB resin that allow the creation of weak interaction and π‐stacking with polyphenolic molecules. Additional observations can be made by the fitting investigation of Weber–Morris and Boyd equations, which serves as a mechanism controller and rate limiter for adsorption. Both the models exhibited significant deviations from the experimental data. This deviation suggests that intraparticle diffusion and film diffusion do not singularly play a dominant role in governing the adsorption process. Anyhow, it is worth noticing that, observing the linearization scatter plots (Figure S13 D,E and S14 E,F, Supporting Information), a discontinuity can be detected at 10 min, defining two different populations of values which show better linearization features if considered independently (see Table 2, R2 bracket values). Thus, it can be supposed that intraparticle and film diffusions are involved in both the adsorption and desorption, with the coexistence of two different kinetics. It is not possible to define if this trend is due to active sites with different nature (energies and accessibility, among others) or to metabolites with different adsorption/desorption features (i.e., polyphenols dimensions). More studies have to be performed on this complex topic, due to the heterogeneous nature of the extract, which, by definition, contains several classes of molecules.

As evident from the graph above presented, the adsorption kinetic suggests that the majority of polyphenols are adsorbed within the first 5 min (not adsorbed value: 27.91%). Based on this analysis, 20 min was chosen as the optimal adsorption time, as the adsorbed polyphenols (0.45%/g_resin_) were considered acceptable compared to the Q_e_ (0.43%/g_resin_). A same comparable trend can be observed for the desorption step, where at 20 min it is possible to reach 1.93%/g_resin_ on the 1.97%/g_resin_ of the Q_e_ extrapolated by the PSO kinetic model.

#### 5.2.4 MS/MS Targeted Characterization of Signature Compounds

As a preliminary characterization of the main fractions (raw extract, HF, and LF), HRM spectroscopy equipped with TOF and exact mass detection and MS/MS fragmentation was used. To achieve a targeted characterization, identification of signature compounds was carried out using commercial standards as reference. The main screened compounds are reported in Table S9, Supporting Information. Concurrently, an analytical protocol for future investigations has been outlined, identifying optimized DP and CE for each molecule, as reported in the same table. Fragmentation optimization is achieved by guided MRM HR infusion and reported in Table S10, Supporting Information. Finally, an approximative comparison between the different composition has been reported in Figure S15 A,B, Supporting Information. Speculations can be made observing the trend of gallic and ellagic acid, as well as procyanidin B1, in relation with the fractionation protocol (see Discussion of Figure S15 A‐B, Supporting Information).

#### 5.2.5 Antioxidant Activity

**Figure 15:**
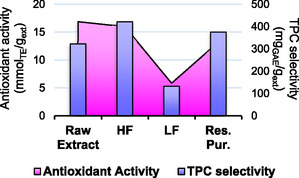
Antioxidant activity and TPC selectivity in main CWW fractions.

After the purification steps, the main fractions were characterized by means of antioxidant activity, by means of DPPH· inhibition. As expected, the antioxidant activity can be related to the polyphenolic content, as depicted in **Figure** **15**.

The HF exhibits an antioxidant activity comparable to that of the raw extract, despite its higher TPC selectivity. This could be attributed to the different reactivity of a tannin‐rich fraction with the *Folin–Ciocalteu* reagent and the DPPH· radical, with respect to low‐weight polyphenols. Regarding to the LF, there is a drop in TPC value (related to the concomitant concentration of salts and sugars), which is correlated with a reduced scavenging activity. However, resin purification (Res. Pur.) enhanced both the parameters, reaching values comparable or even higher to the raw extract. This result further substantiates the efficacy of the purification protocol performed on LF, enabling the recovery of actives from a by‐product for HF production process.

#### 5.2.6 Enzymatic Assays

**Figure 16:**
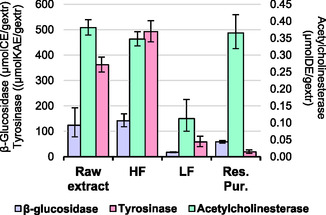
Inhibition potential of different fractions (raw extract, HF, LF, and Res. Pur.) on β‐glucosidase, acetylcholinesterase, and tyrosinase.

To comprehensively explore the diverse applications of chestnut extracts, a series of enzymatic inhibition assays were conducted, targeting enzymes linked to major health and industrial concerns. These enzymes include AChE, relevant to neurodegenerative disorders, β‐glucosidase, associated with antiproliferative effects and antiviral potential, and tyrosinase, implicated in melanogenesis and enzymatic browning. The obtained results are displayed in **Figure** **16** and can help to shed light on activity distribution according to the different fractionations.

β‐Glucosidase plays a critical role in the hydrolysis of β‐1,4‐glycosidic bonds, primarily converting cellobiose into glucose. Additionally, it is involved in glycoprotein processing, which contributes to the development of viral envelopes.^[^ ^69^ ^]^ Given these dual roles, β‐glucosidase inhibition holds therapeutic potential for the treatment of type 2 diabetes and viral infections.^[^ ^70^ ^]^ The enzymatic inhibition assays identified HF as the most active fraction. The observed increase in castanospermine‐equivalent activity within this fraction, along with a corresponding decrease in activity in the low‐molecular‐weight fraction relative to the initial extract prior to fractionation, suggests that tannins play a primary role in the observed inhibitory effects

Natural acetylcholinesterase inhibitors, such as polyphenols found in plants, show promise for Alzheimer's treatment, offering safer alternatives to toxic synthetic inhibitors like carbamates and organophosphates.^[^ ^71^ ^]^ AChE inhibition was observed across all tested fractions, except for LF. However, the purification of this low‐molecular‐weight fraction using resin significantly enhanced its TPC, ultimately restoring its inhibitory activity to levels comparable to the other fractions. These findings align with existing literature, which underscores the potent inhibitory effects of polyphenols irrespective of molecular size, with both tannin‐rich and low‐weight phenols‐rich fractions exhibiting high activity.^[^ ^72^, ^73^ ^]^ Further studies have confirmed the AChE inhibitory potential of natural polyphenols, with in vitro and in vivo tests on rats demonstrating their bioactivity and relevance for neurodegenerative disease treatment.^[^ ^74^ ^]^

Tyrosinases are a copper‐based class of enzymes involved in the hyperpigmentation or melanogenesis of human skin and in the enzymatic browning of fruit and vegetables.^[^ ^75^ ^]^ Inhibitors of these enzymes can be applied in both the cosmetic field as anti‐melogenesis skincare products and in the food industry as preservatives.^[^ ^76^ ^]^ In literature, several studies are reported on the potential inhibitor activity of tannin toward tyrosinases tested both in vitro and in intracellular conditions.^[^ ^77^, ^78^ ^]^ In our study, the most active fraction has been proved to be HF, with an increase of 25% in the inhibitor activity compared to the raw fraction. Interestingly, the two small molecular fractions, namely LF and Res. Pur., demonstrated almost an absence of activity toward tyrosinase inhibition.

#### 5.2.7 Antibacterial Activity

All the extracts displayed antibacterial effects showing different activity levels against microorganisms tested, while no effect was detected in the negative controls. The results of disk diffusion tests are reported in **Table** **3**. The LF and the Res. Pur. exhibited similar results, with antibacterial effects only against *Staphylococcus aureus* and *Pseudomonas aeruginosa*. On the contrary, the raw extract and, particularly, the HF, rich in tannins, showed antibacterial activity against all microorganisms, except *Listeria monocytogenes*. Among the microorganisms used to evaluate antibacterial activity, *Staphylococcus* *aureus* was the most sensitive to the extracts, according to the results obtained in several studies.^[^ ^79^, ^80^ ^]^ These studies reported a greater effect of tannin‐rich extracts, particularly tannic acid and its derivatives, on gram‐positive microorganisms probably due to the characteristic of the cell wall.^[^ ^81^, ^82^ ^]^ However, the absence of activity against *Listeria monocytogenes* (a gram‐positive microorganism) by the extracts, combined with the antibacterial activity against gram‐negative microorganisms (e.g., *Pseudomonas aeruginosa*), generally less or minimally sensitive to tannic acid and its derivatives, suggests that the extracts may contain a mixture of tannins with differing and broader‐spectrum activities. This hypothesis is in accordance with condensed/hydrolyzable ratios, investigated in the **Tannin Composition section**.

**Table 3:** Results of disk diffusion test reported as the average (±standard deviation) of the halos diameter measured (mm). Tested samples: raw extract, HF, light fraction (LF), resin purified (res. Pur), tannic acid (TA), gallic acid (GA), negative control (C‐); ‐: no effect.

Microorganism	Raw extract	HF	LF	Res. Pur.	TA	GA	C‐
*E. coli*	8.5 ± 0.7	9.5 ± 0.7	–	–	–	–	–
*S. typhimurium*	8.0 ± 0.0	8.0 ± 0.0	–	–	–	–	–
*L. monocytogenes*	–	–	–	–	10.0 ± 0.0	–	–
*S. aureus*	14.0 ± 0.0	11.0 ± 1.4	9.0 ± 0.0	10.0 ± 0.0	15.5 ± 0.7	8.0 ± 0.0	–
*P. aeruginosa*	12.0 ± 0.0	10.0 ± 0.0	7.0 ± 0.0	9.0 ± 1.4	11.5 ± 0.7	–	–

These preliminary data highlight the need to deep the potential application of these extracts in various industrial chains. In food production, these compounds could be used to control the growth of pathogenic or spoilage bacteria.

## 6 Conclusion

This study demonstrates the potential of MASWE and green downstream techniques in valorizing CWW for sustainable applications. By optimizing extraction conditions using CCD, we successfully balanced low‐weight polyphenols and tannic fractions, maximizing the recovery of bioactive compounds. The cascade membrane filtration and resin adsorption protocols further refined the extracts, allowing for targeted enrichment of polyphenolic fractions with enhanced antioxidant and antimicrobial properties. Among these, the tannin‐enriched fraction exhibited the highest bioactivity and was effectively integrated into a PVA‐based biopolymer, demonstrating promising physicochemical and functional properties. This work highlights an innovative, eco‐friendly approach to biowaste valorization, reinforcing the principles of the circular economy and green extraction by means of a fractionation approach. Since biomass extractions yield complex mixtures, it is crucial to maximize the potential of each component (or group of components). Fractionation plays a crucial role in biomass valorization by enabling the efficient separation of metabolite classes, each characterized by distinct features and potential activities. This approach enhances industrial applicability, optimizing resource utilization while aligning with circular economy principles. The findings suggest potential applications for these bioactive extracts in biopolymers, functional materials, and sustainable industries, paving the way for further research on scalable, water‐efficient extraction strategies to maximize waste‐derived resources while minimizing environmental impact.

## Conflict of Interest

The authors declare no conflict of interest.

## Supporting information

Supplementary Material

## Data Availability

The data that support the findings of this study are available from the corresponding author upon reasonable request.
