# Supplementary material for: Green Extraction and Fractionation of Chestnut Wood Waste: A Sustainable Pathway to Biopolymers and Antimicrobial Solutions
Source: ChemSusChem. 2025 Jul 31;18(18):e202500498. doi: 10.1002/cssc.202500498 (PMC12456377; doi:10.1002/cssc.202500498)
Supplement: Supplementary file 1 — Supplementary Material [file CSSC-18-e202500498-s001.pdf]

Green Extraction and Fractionation of Chestnut Wood Waste: A Sustainable Pathway to Biopolymers and Antimicrobial Solutions

Clelia Aimone,[a] Giorgio Capaldi,[a] Salah Chaji,[a] Emanuela Calcio Gaudino,[a] Anastasia Anceschi,[b] Alessia Patrucco,[b] Silvia Bonetta,[c] Manuela Macrì,[c] Giorgio Grillo,*[a] Giancarlo Cravotto[a]

[a] C. Aimone, G. Capaldi, S. Chaji, Prof. E. Calcio Gaudino, Dr. G. Grillo, Prof. G. Cravotto,
Department of Drug Science and Technology
University of Turin
Via P. Giuria 9, 10125 Turin, Italy
E-mail: giorgio.grillo@unito.it *

[b] Dr. A. Anceschi, Dr. A. Patrucco
Institute of Intelligent Industrial Technologies and Systems for Advanced Manufacturing (STIIMA)
Italian National Research Council (CNR)
Corso G. Pella 16, 13900, Biella (BI), Italy

[c] Prof. Silvia Bonetta, M. Macrì
Department of Life Sciences and Systems Biology
University of Turin
Via Accademia Albertina 13, 10123, Torino, Italy

Supporting information

Table S1: Abbreviation List.

Abbreviation	Explanation
ADS	Adsorption
ATR	Attenuated Total Reflectance
CCD	Central composite design
CWW	Chestnut wood waste
DES	Desorption
DF	Diafiltration
DSC	Differential Scanning Calorimetry
GAE	Gallic acid equivalent
Gly	Glycerol
HF	Heavy fraction
ItA	Itaconic anhydride
L/S	Liquid to solid
LF	Light fraction
MASWE	Microwave-assisted subcritical water
MF	Mixed fraction
MW	Microwave

MWCO	Molecular weight cut-off
NF	Nanofiltration
PF	Pectin-rich fraction
PFO	Pseudo-first order
PNF	Permeate nanofiltration
PSO	Pseudo-second order
PUF	Permeate ultrafiltration
PVA	Polyvinyl Alcohol
RNF	Retentate nanofiltration
RT	Room temperature
RUF	Retentate ultrafiltration
SB	Sepabeads resin
TGA	Thermogravimetric analysis
TPC	Total polyphenolic content
TSC	Total sugar content
UF	Ultrafiltration
US	Ultrasounds

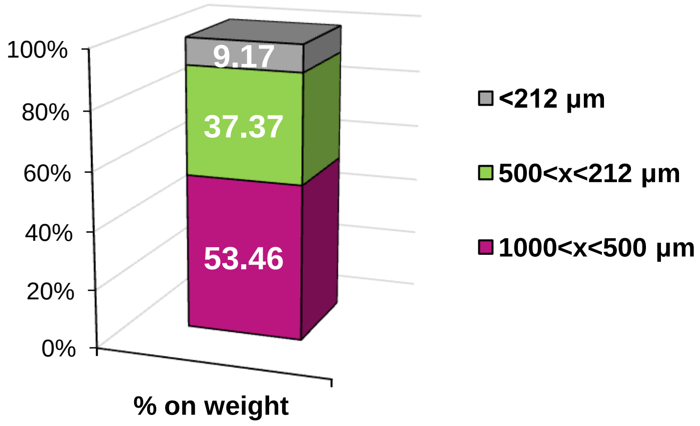

Figure S1: Particle size distribution of extracted CWW.

Table S2: Range and variables used for the CDD for the optimization of microwave-assisted subcritical water extraction (T: temperature; L/S: liquid to solid ratio).

Factor	Time (min)	T (°C)	L/S
-1.6818	3.1	79.5	3.2
-1	14.0	104.0	10.0
0	30.0	140.0	20.0
+1	46.0	176.0	30.0
+1.6818	56.9	200.5	36.8

Table S3: Experimental parameters of the CDD for the optimization of microwave-assisted subcritical water extraction and the responses for the dry yield (T: temperature; L/S: liquid to solid ratio).

Run	Time (min)	T (°C)	L/S	Dry Yield (%)
1	14.0	104.0	10.0	14.44
2	46.0	104.0	10.0	14.44
3	14.0	176.0	10.0	26.30
4	46.0	176.0	10.0	22.40
5	14.0	104.0	30.0	17.25
6	46.0	104.0	30.0	15.84
7	14.0	176.0	30.0	29.04
8	46.0	176.0	30.0	27.30
9	3.1	140.0	20.0	16.04
10	56.9	140.0	20.0	20.24
11	30.0	79.5	20.0	13.96
12	30.0	200.5	20.0	18.60
13	30.0	140.0	3.2	12.51
14	30.0	140.0	36.8	21.17
15	30.0	140.0	20.0	18.84
16	30.0	140.0	20.0	19.48
17	30.0	140.0	20.0	19.20
18	30.0	140.0	20.0	19.64

Membrane Filtration Detailed Procedure. To start the fractionation, *approx*. 3 L of the optimized extract were first processed with UF_{1000}, after 76 min, 2.3 L of permeate (PUF_{1000} 1) were collected and 2.3 L of deionized water have been added to the tank to diafiltrate the retentate. After 79 min 2.3 L of permeate PUF_{1000} 2–DF were separated. RUF_{1000} was collected, an aliquot was dried, and the membrane washed with deionized water to enhance the recovery of the material.

PUF_{1000} 1 were processed with $NF_{150\text{-}300}$. In 5.27 min 1800 mL of $PNF_{150\text{-}300}$ 1 were recovered. With the PUF_{1000} 2–DF the diafiltration of the retentate was carried out, and in 8 min 2.3 L of $PNF_{150\text{-}300}$ 2–DF were separated. $RNF_{150\text{-}300}$ was collected, an aliquot was dried, and the membrane washed with deionized water to enhance the recovery of the material.

RUF_{1000} was diluted with 1 L of water (for minimal volume requirements) and processed with UF_{5000}. After the separation of 1 L of PUF_{5000} 1, 1 L of deionized water was added to diafiltrate the retentate and 1 L of PUF_{5000} 2–DF was separated. RUF_{5000} was collected (named heavy fraction, HF), an aliquot was dried, and the membrane washed with deionized water to enhance the recovery of the material. $RNF_{150\text{-}300}$ was diluted with 950 mL of PUF_{5000} 1 and processed with $NF_{600\text{-}800}$. In 8 min 950 mL of $PNF_{600\text{-}8000}$ 1 were separated and with 1 L of PUF_{5000} 2–DF the diafiltration of the retentate were carried out, collecting 1 L of $PNF_{600\text{-}8000}$ 2–DF. $RNF_{600\text{-}800}$ was collected, an aliquot was dried, and the membrane washed with deionized water to enhance the recovery of the material. The whole PNF $_{600\text{-}800}$ unified was inserted into the tank and processed with $NF_{150\text{-}300}$. From 1850 mL of feed, 1530 mL of $PNF_{150\text{-}300}$ final have been separated. $RNF_{150\text{-}300}$ final (namely light fraction, LF) was collected, an aliquot was dried, and the membrane washed with deionized water to enhance the recovery of the material.

Table S4: Experimental parameters of the CDD for the optimization of the adsorption process and the responses for the TPC (T: temperature; L/S: liquid to solid ratio).

Run	T (°C)	L/S	Non-adsorbed polyphenols (%)
1	25.00	25.00	18.85
2	25.00	100.00	24.96
3	45.00	25.00	23.51
4	45.00	100.00	29.20
5	35.00	62.50	23.58
6	35.00	62.50	24.88
7	35.00	62.50	24.63
8	35.00	62.50	24.34
9	35.00	115.53	27.43
10	35.00	9.47	19.43
11	49.14	62.50	27.31
12	20.86	62.50	21.91

Table S5: Experimental parameters of the CDD for the optimization of the desorption process and the responses for the percentage of adsorbed polyphenols (T: temperature; L/S: liquid to solid ratio).

Run	T (°C)	L/S	Desorbed polyphenols (%)
1	25	15	95
2	25	80	98
3	45	15	89
4	45	80	83
5	35	47.5	92
6	35	47.5	96
7	35	47.5	94
8	35	47.5	96
9	35	93.46	85
10	35	1.54	34
11	49.14	47.5	90
12	20.86	47.5	99

Table S6*:* Kinetic model: Equation and Linearization.

**Model**	**Equation**	**Linearization**
Pseudo-first order (PFO)	$Q_t = Q_e(1 - e^{-kt})$	$\ln(Q_e - Q_t) = lnQ_e - k_1 \cdot t$
Pseudo-second order (PSO)	$Q_t = \frac{k_2 \cdot {Q_e}^2 \cdot t}{1 + k_2 Q_e t}$	$\frac{t}{Q_t} = \frac{1}{k_2 \cdot {Q_e}^2} + \frac{t}{Q_e}$
Peleg (Hyperbolic)	$Q_t = \frac{t}{k_1 + k_2 t}$	$\frac{1}{Q_t} = k_1 \cdot \frac{1}{t} + k_2$
Power law	$Q_t = B \cdot t^n$	$lnQ_t = lnB + n \cdot lnt)$
Weber-Morris (*Intraparticle diffusion*)	$Q_t = \ k_i \cdot t^{\frac{1}{2}} + c$	Already Linear
Boyd (*Liquid film diffusion*)	$\frac{Q_t}{Q_e} = \ e^{-kt+c}$	$\ln\left(1 - \frac{Q_t}{Q_e}\right) = -k_f \cdot t + c$
Elovich (*Specific chemical interactions*)	$Q_t = \frac{1}{\beta}\ln\,(1 + \alpha\beta t)$	$Q_t = \frac{1}{\beta}\ln(\mathrm{t}) + \frac{1}{\beta}(\alpha\beta)$
Extracted species at time “t”	$Q_t = TPC\ Yield\ (\%)\ at\ time\ \text{"t"}$	
Extracted species at equilibrium	$Q_e = TPC\ Yield\ (\%)\ at\ equilibrium$	
Adsorbed/desorbed species at time “t”	$Q_t = \frac{(C_0 - C_t) \cdot V}{m}$	
Adsorbed/desorbed species at equilibrium	$Q_e = \frac{(C_0 - C_e) \cdot V}{m}$	

V: Processed volume; m: Resin mass; C_0: Species concentration at time 0; C_t: Species concentration at time t; C_e: Species concentration at equilibrium.

A

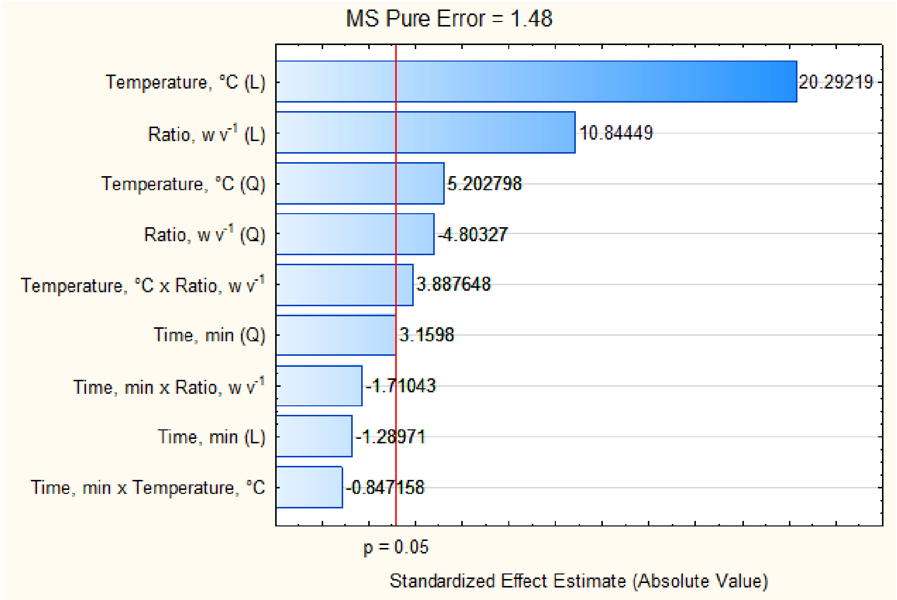

B

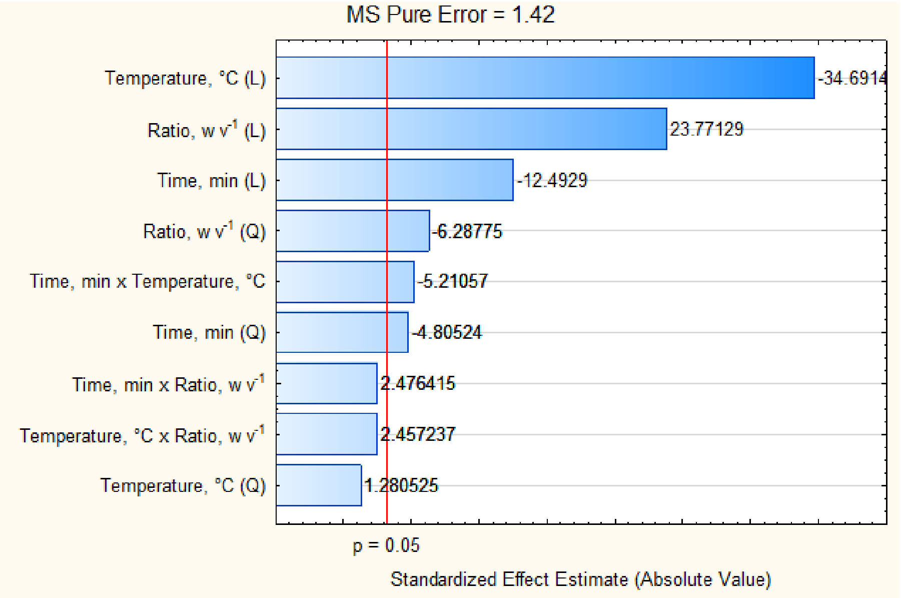

Figure S2: Pareto charts of standardized effects obtained for the optimization of the extraction process of low-weight polyphenols (A) and tannins (B).

Table S7: Polyphenols distribution (expressed in %) for three different L/S ratio.

L/S	Low-weight polyphenols (%)	Tannins (%)
10	45.09	54.91
20	43.13	56.87
30	45.71	54.29

***Figure S3*:** Linear regression for extraction kinetics. A: PFO; B: PSO; C: Peleg (*Hyperbolic*); D: Power law; E: Weber-Morris (*Intraparticle diffusion*); F: Boyd (*Liquid film diffusion*); G: Elovich (*Specific chemical interactions*).

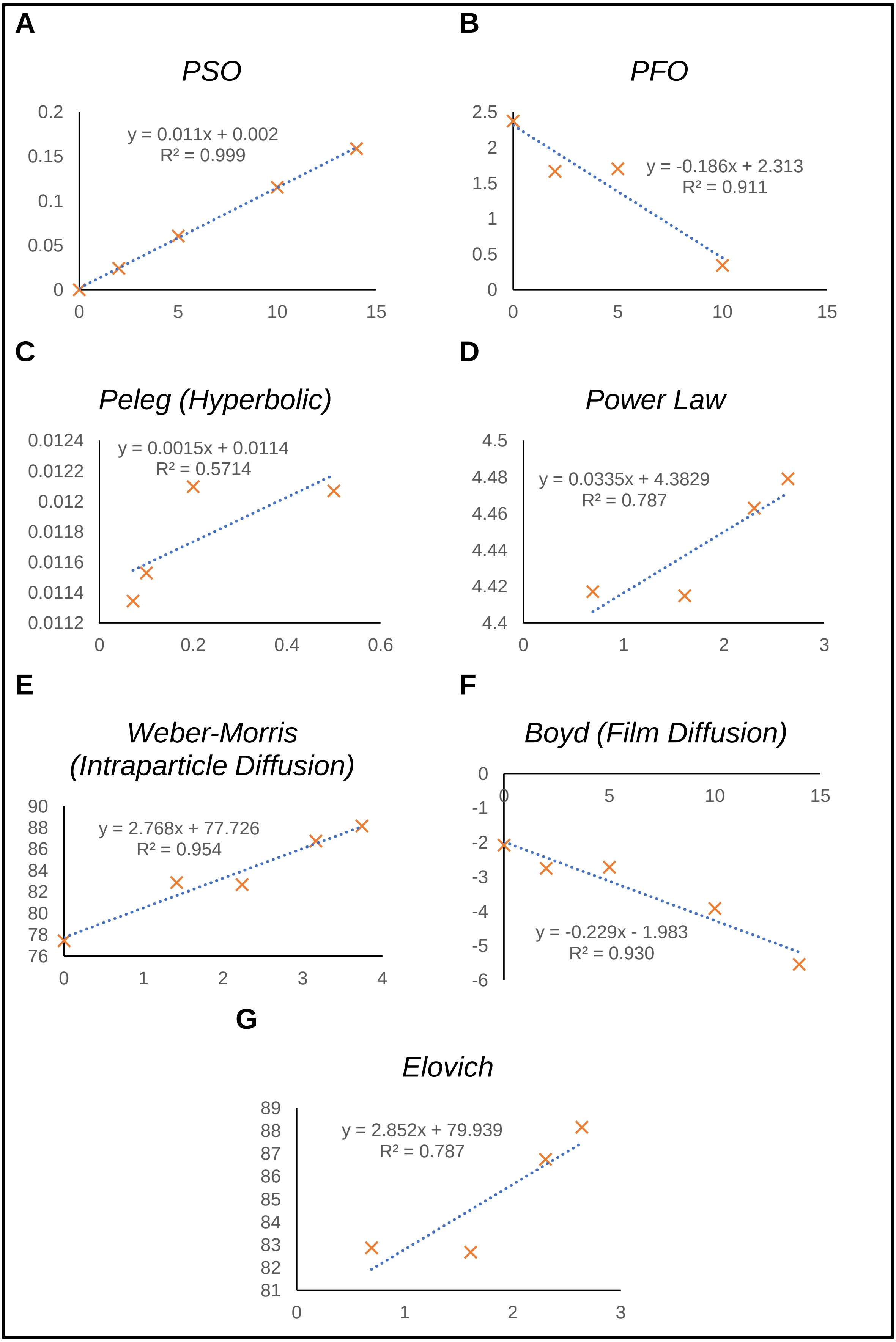

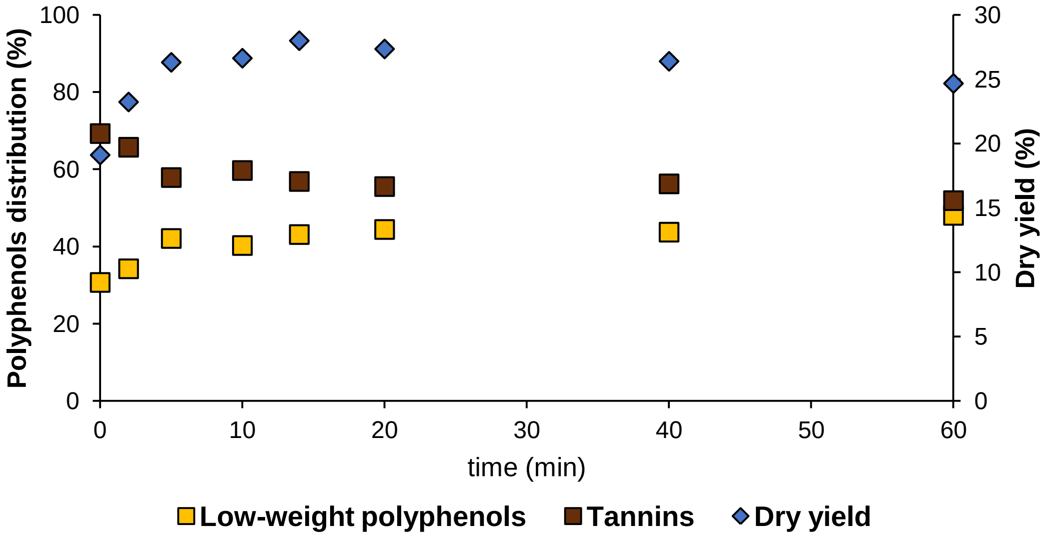

Figure S4: Kinetic study, based on the optimal conditions (T: 176 °C; L/S: 20).

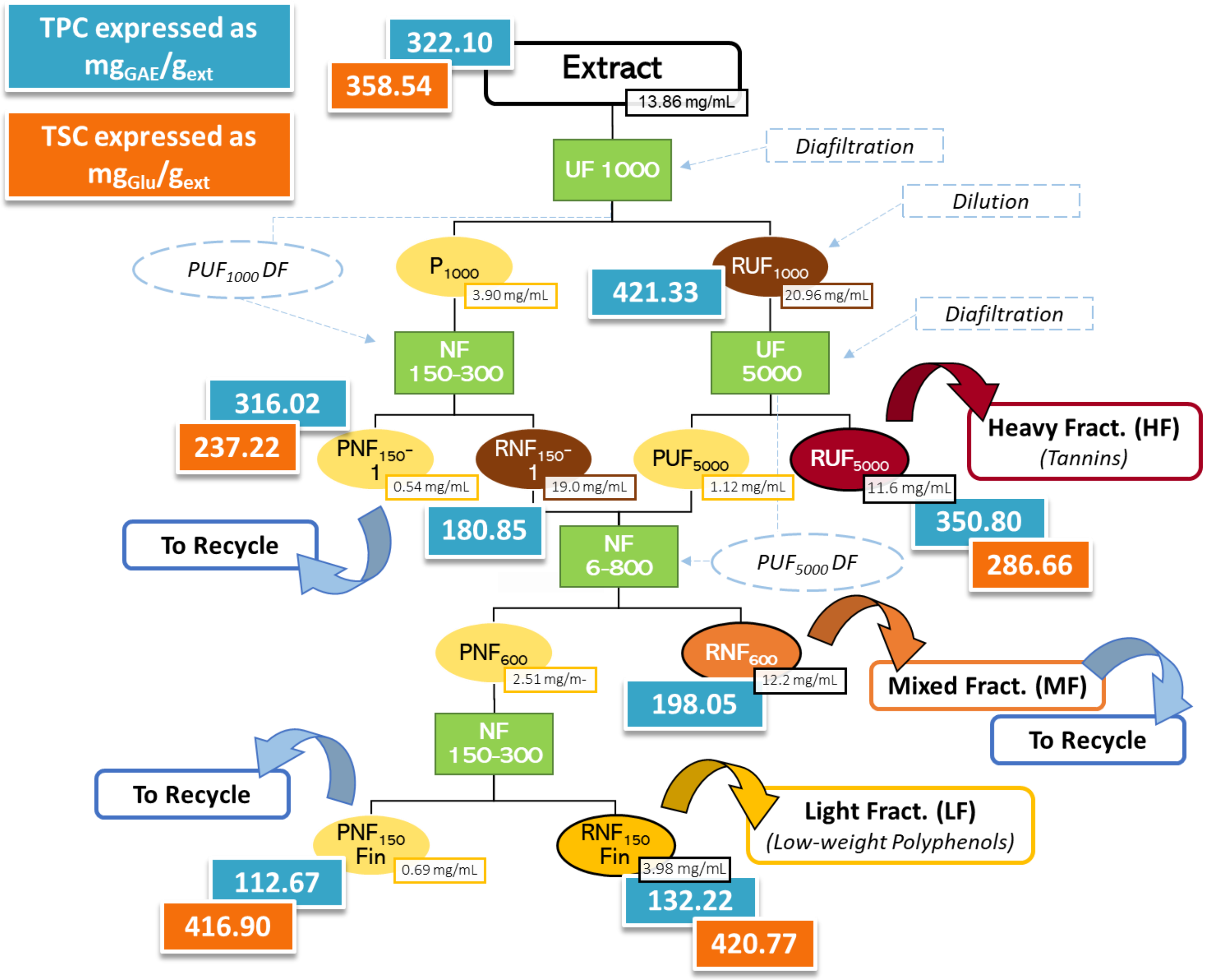

Figure S5: Membrane filtration results: TPC, TSC and concentrations.

Table S8: Composition of the prepared biopolymers. All formulations were prepared using 2 mL of water and where not reported differently, 100 mg of PVA. The percentages of the other components are reported as % normalized on PVA, Pectin enriched extract or their combination.

PVA - BASED		
Sample 1	***Sample 2***	***Sample 3***
45% HF	45 % HF	45 % HF
5 % ItA	5 % ItA	5 % ItA
0 % Gly	10 % Gly	20 % Gly
**FRACTURED**	**RIGID**	**NOT UNIFORM**
Sample 4	***Sample 5***	***Sample 6***
40 % HF	35 % HF	40 % HF
5 % ItA	5 % ItA	0 % ItA
20 % Gly	20 % Gly	0 % Gly
**UNIFORM/ELASTIC**	**RIGID**	**FRACTURED**
PVA/PECTIN – BASED		
Sample 7	***Sample 8***	***Sample 9***
25 mg PVA	50 mg PVA	75 mg PVA
75 mg Pectin enriched extract	50 mg Pectin enriched extract	25 mg Pectin enriched extract
40 % HF	40 % HF	40 % HF
5 % ItA	5 % ItA	5 % ItA
20 % Gly	20 % Gly	20 % Gly
**FRACTURED**	**FRACTURED**	**UNIFORM /ELASTIC**
PECTIN – BASED [a]		
Sample 10	***Sample 11***	
40 % HF	40 % HF	
0 % ItA	5 % ItA	
20 % Gly	20 % Gly	
**RIGID**	**FRACTURED**	
REFERENCES - PVA BASED		
Reference1	***Reference2***	
40 % HF	40 % HF	
0 % ItA	5 % ItA	
0 % Gly	0 % Gly	
**RIGID**	**RIGID**	

[a]: Pectin enriched extract adopted in complete substitution of PVA (100 mg). Other components percentages are normalized accordingly.

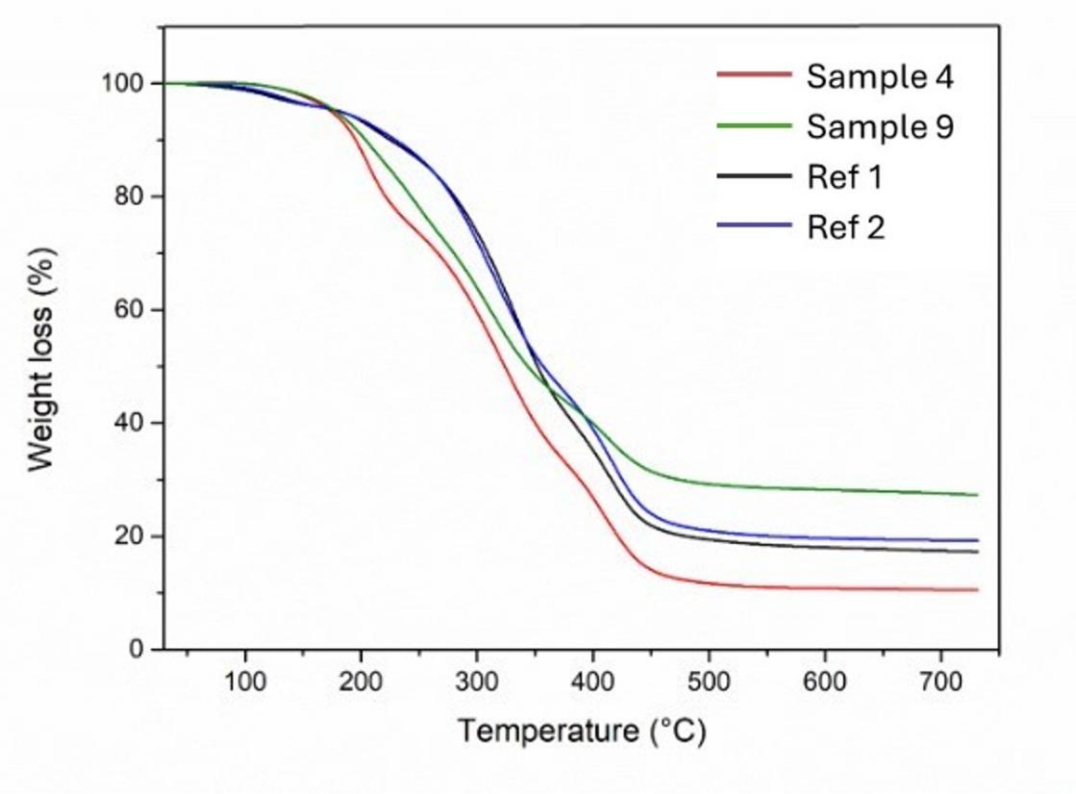

Figure S6**:** TGA analysis of sample 4, sample 9, reference 1 (ref 1) and reference 2 (ref 2)

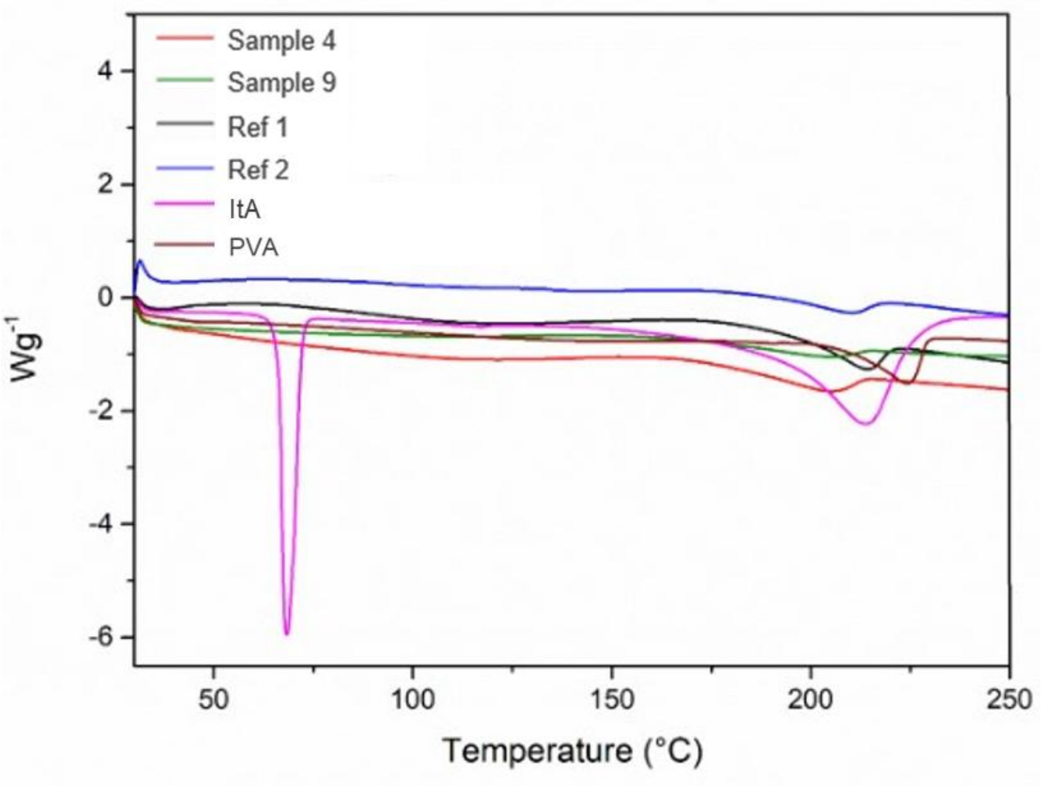

Figure S7: DSC of sample 4, sample 9, reference 1 and reference 2 compared with itaconic anhydride and PVA

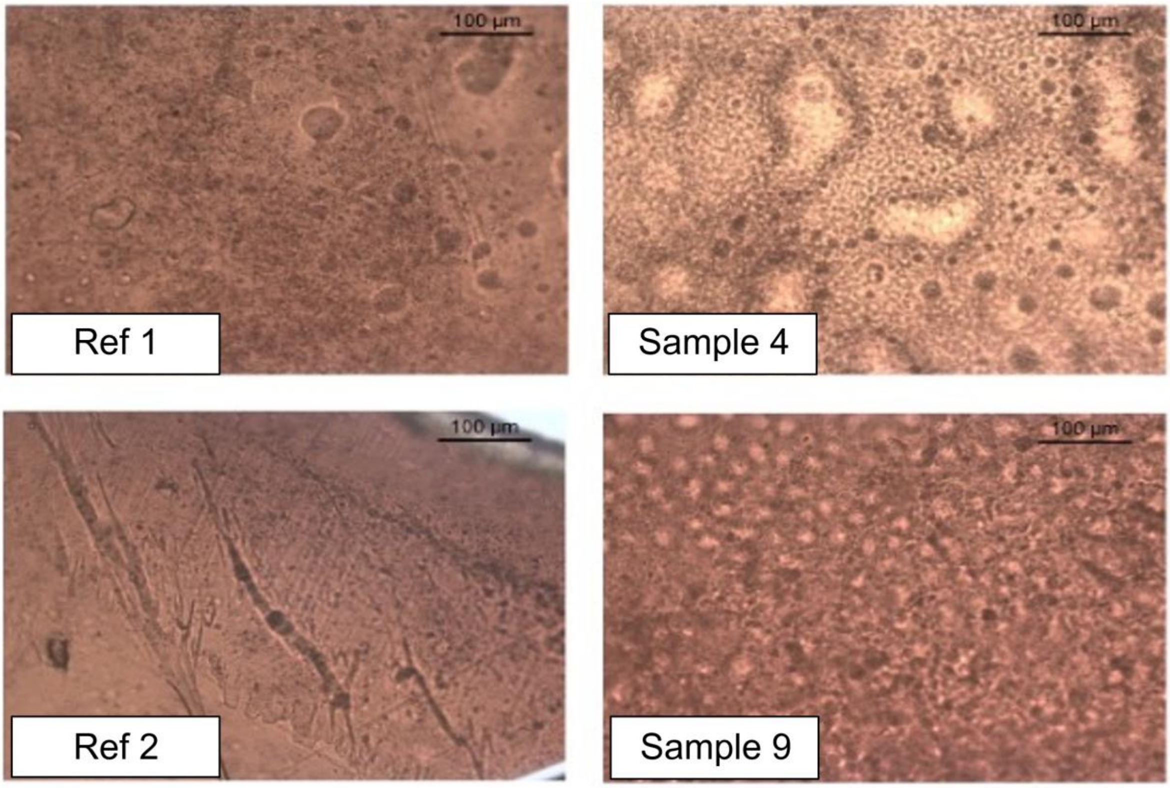

Figure S8: Optic microscopy pictures of sample 4, sample 9, reference 1 (ref 1) and reference 2 (ref 2)

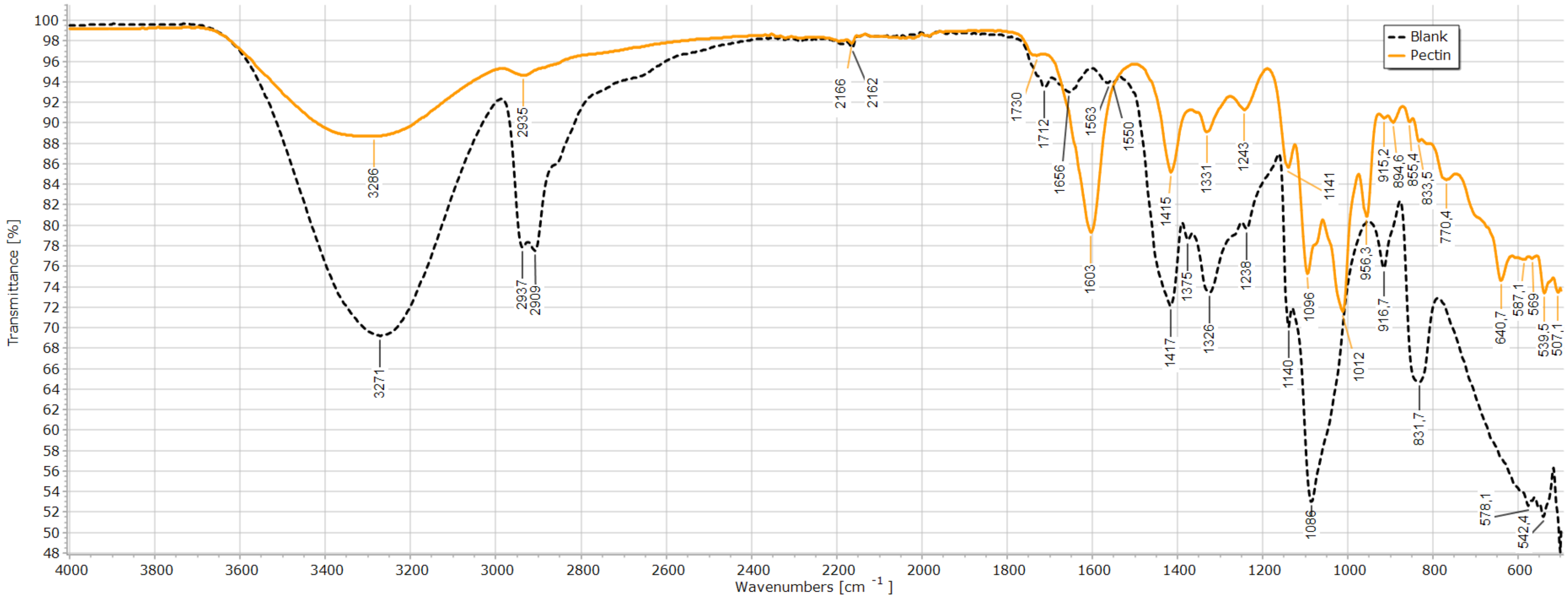

Figure S9: Attenuated Total Reflectance (ATR), PVA and pectin fingerprints.

Discussion:

Pectin: The large band between 3000 and 3700 cm^{-1}, belongs to -OH stretching modes, whilst the near signal at 2935 cm^{-1} refers to C-H stretching (vibrations of CH_2 and CH_3 groups, commonly found in the methyl esters and acetyl groups within pectin). The band at 1730 cm^{-1} can be assigned at C=O stretching (methyl ester group and undissociated carboxylic acid). Furthermore, the ATR presents a band at around 1656 cm^{-1} attributable to the stretching of nonesterified =O groups, as well as other bands typical of pectin (approximately between 900 and 1450 cm^{-1}). In particular is possible to detect signals at 1415 cm^{-1} (bending of -O-H bonds), 1326 cm^{-1} (vibration of CH_2 groups), 1140 cm^{-1} and 1086 cm^{-1} (both C-O-C stretching of glycosidic linkages). The region below 900 cm^{-1} contains weaker bands related to various C-O-C bridge vibrations and other ring vibrations coupled with C-OH bending.[1]

Blank: The registered spectrum corresponds to PVA, as demonstrated by the following diagnostic bands assignations. Broad band with max in 3271 cm^{-1} is related to (O-H) stretching vibration from the intermolecular and intramolecular hydrogen bonds. The two bands at 2937 and 2909 cm^{-1} correspond to the asymmetric and symmetric stretching vibrations of methylene (CH_2), respectively. The peaks at 1712 cm^{-1}, 1656 cm^{-1}, and 1563 cm^{-1} have been related to the stretching vibrations of the (C=O) and (C–O) bonds present in the residual acetate units.[2] 1656 cm^{-1} band has also been assigned to absorbed water. The peaks at 1417 cm^{-1} and 1327 cm^{-1} has been attributed respectively to bending vibrations of hydroxyl groups and to wagging of (C–H). [3] Interestingly, the band at 1140 cm^{-1} is ascribed to (C–O) stretching vibrations in C–OH groups of the crystalline polymer phase as well as to (C–C) stretching vibrations of the carbon framework of the polymer chain in the crystalline phase. The intense signal at 1086 cm^{-1} corresponds to the (C–O) stretching vibrations. [4] The band at 916 cm^{-1} to CH_2 rocking vibration and 831 cm^{-1} to the (C–C) stretching and (C–H) out-of-plane vibrations. It worth notice that no particular signal belonging to the Gly addition can be detected, likely due to the low additive content. [5]

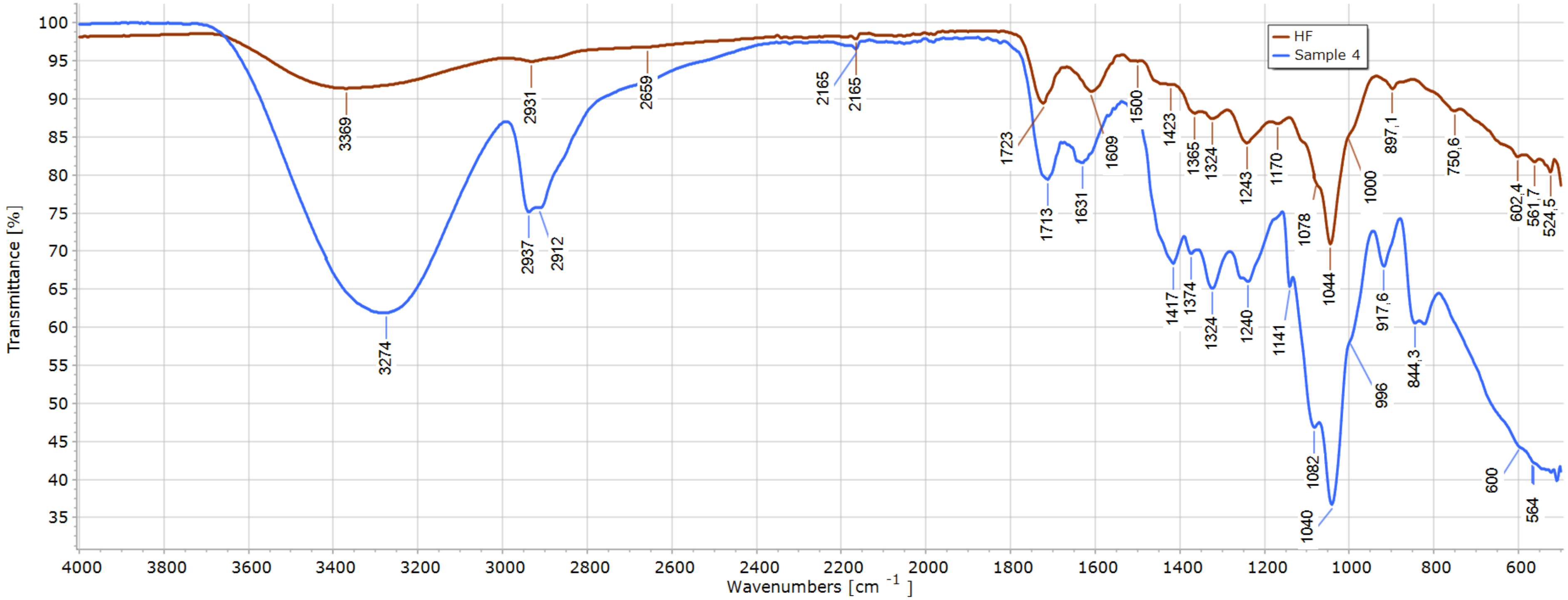

Figure S10: Attenuated Total Reflectance (ATR), direct comparison between HF and Sample 4.

Discussion:

HF: The typical broad band among 3369 cm⁻¹ can be ascribed at -OH stretching and aromatic CH stretching, whilst the shoulder at 2931 cm⁻¹ can be assigned to aliphatic CH_3 and CH_2 stretching. Other bands that can be put in relation with aromatic components are 1723 cm⁻¹, 1609 cm⁻¹, 1500 cm⁻¹ and 1423 cm⁻¹, which are respectively respondent to phenyl ester linkage vibrations, aromatic C-C sym stretching, ring in-plane bending and aromatic C-C antisym stretching. Conversely, signals at 1243 cm⁻¹, 1170 cm⁻¹ and 1044 cm⁻¹ are assigned to aliphatic C–O sym stretching, aliphatic C–O stretching (ether or alcohol functionalities). The shoulder at 1078 cm⁻¹ match with (C=O) stretching, indicating the presence of carbonyl groups, likely from ester or carboxylic acid functionalities. Lastly, low intensity signals between 897 cm⁻¹ and 750 cm⁻¹ can be related to out-of plane bending vibrations of (C−H) bonds and specific bending modes in the biopolymer structure. [6]

Sample 4: The spectra registered for sample 4, which has been made without the addition of pectin has been selected as the clearest to point out the interaction between the loading of HF fraction and the polymeric network of PVA/Gly. Several bands appear to be maintained, presenting a shift that assesses an effective interaction between the loading and the PVA framework. As example: 1713 cm⁻¹, 1631 cm⁻¹, 1374 cm⁻¹, 1040 cm⁻¹. Other signals are diagnostic for PVA (see Discussion of **Figure S9**) with a related shift and will be listed in the Discussion of **Figure S11**.

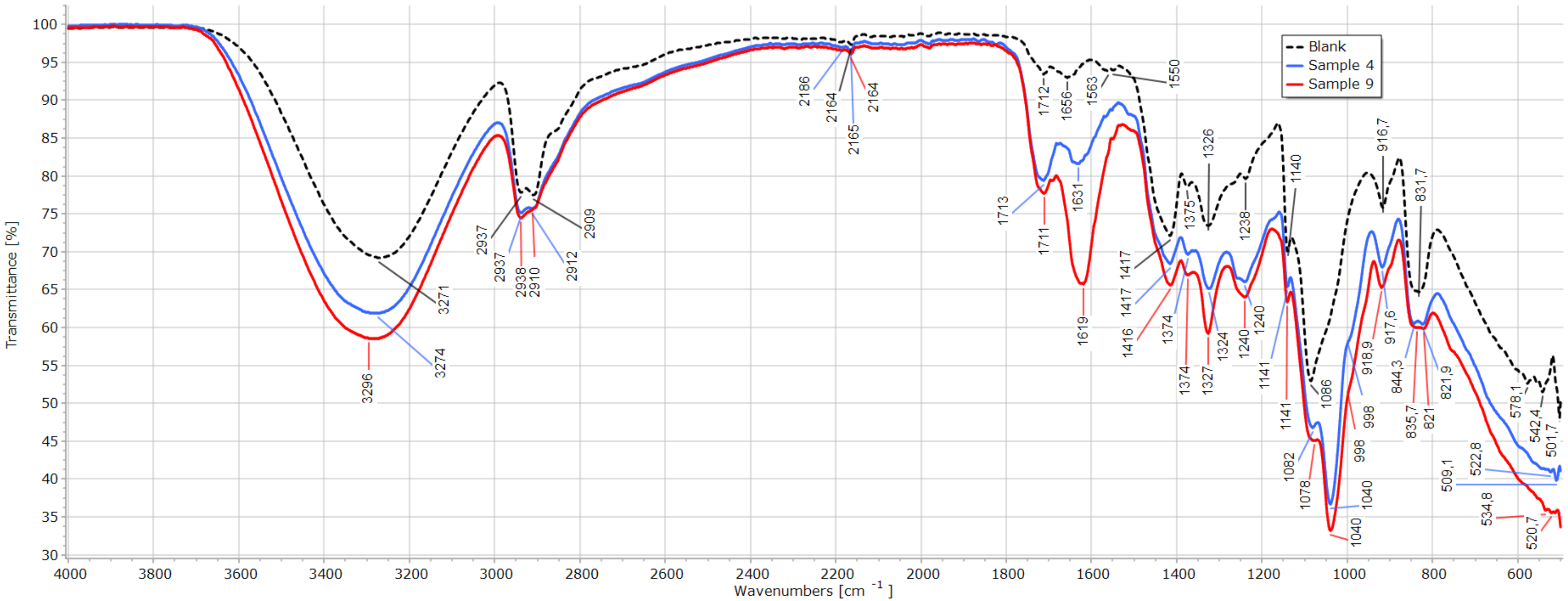

Figure S11: Attenuated Total Reflectance (ATR), direct comparison between blank and samples 4 and 9.

Discussion:

The Figure point put the similarities between the sample 4 and 9 and the blank adopted as a reference for PVA/Gly. As clearly visible the main bands of the PVA, listed in the Discussion of **Figure S9**, are present in both the samples, with partial shifts. It worth notice of course the presence of pectin diagnostics signals in case of sample 9. The peculiar shift of the broad band, as reported in literature, can be related to hydrogen bond formation and interaction of the OH groups of the polymer with the loading. [7,8]. It worth notice that the signal belonging to PVA crystallinity (1140 cm^{-1}) is retained in both the samples, likely helping the mechanical features of the final product. The most intense signal of sample 4 and 9 at 1040 cm^{-1} can be referred to HF aliphatic C–O antisym stretching (indicating ether or alcohol functionalities), associated to the sym stretching at 1240 cm^{-1}. The aromatic contribute of the bioactive loading can be evidenced by two others intense bands 1713 cm^{-1}/1631 cm^{-1} (sample 4) and 1711 cm^{-1}/1619 cm^{-1} (sample 9) related to aromatic C-C sym stretching and phenyl ester linkage vibration.

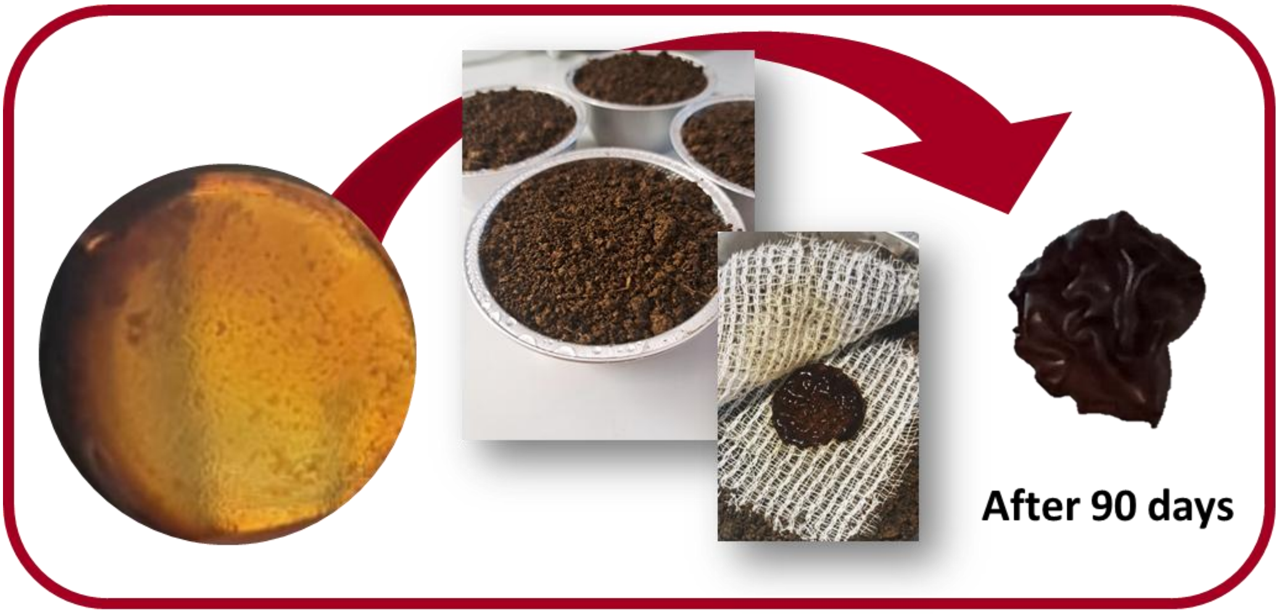

Figure S12: Soil burial images throughout the treatment time.

Figure S13: Linear regression for adsorption kinetics. A: PSO; B: Peleg (*Hyperbolic*); C: Power law; D: Weber-Morris (*Intraparticle diffusion*); E: Boyd (*Liquid film diffusion*); F: Elovich (*Specific chemical interactions*).

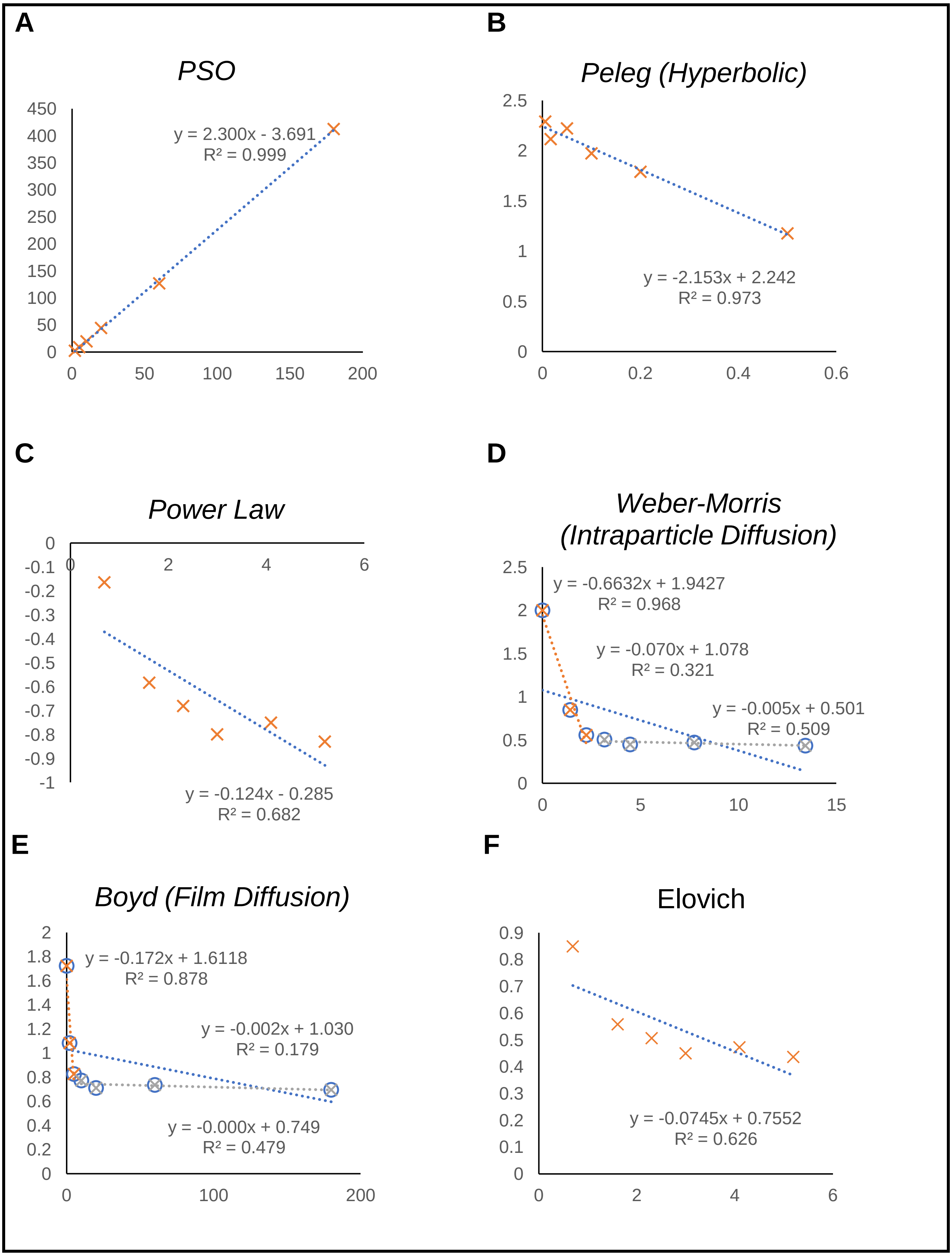

Figure S14: Linear Regression for Desorption Kinetics. A: PFO; B: PSO; C: Peleg (*Hyperbolic*); D: Power Law; E: Weber-Morris (*Intraparticle Diffusion*); F: Boyd (*Liquid Film Diffusion*); G: Elovich (*Specific Chemical Interactions*).

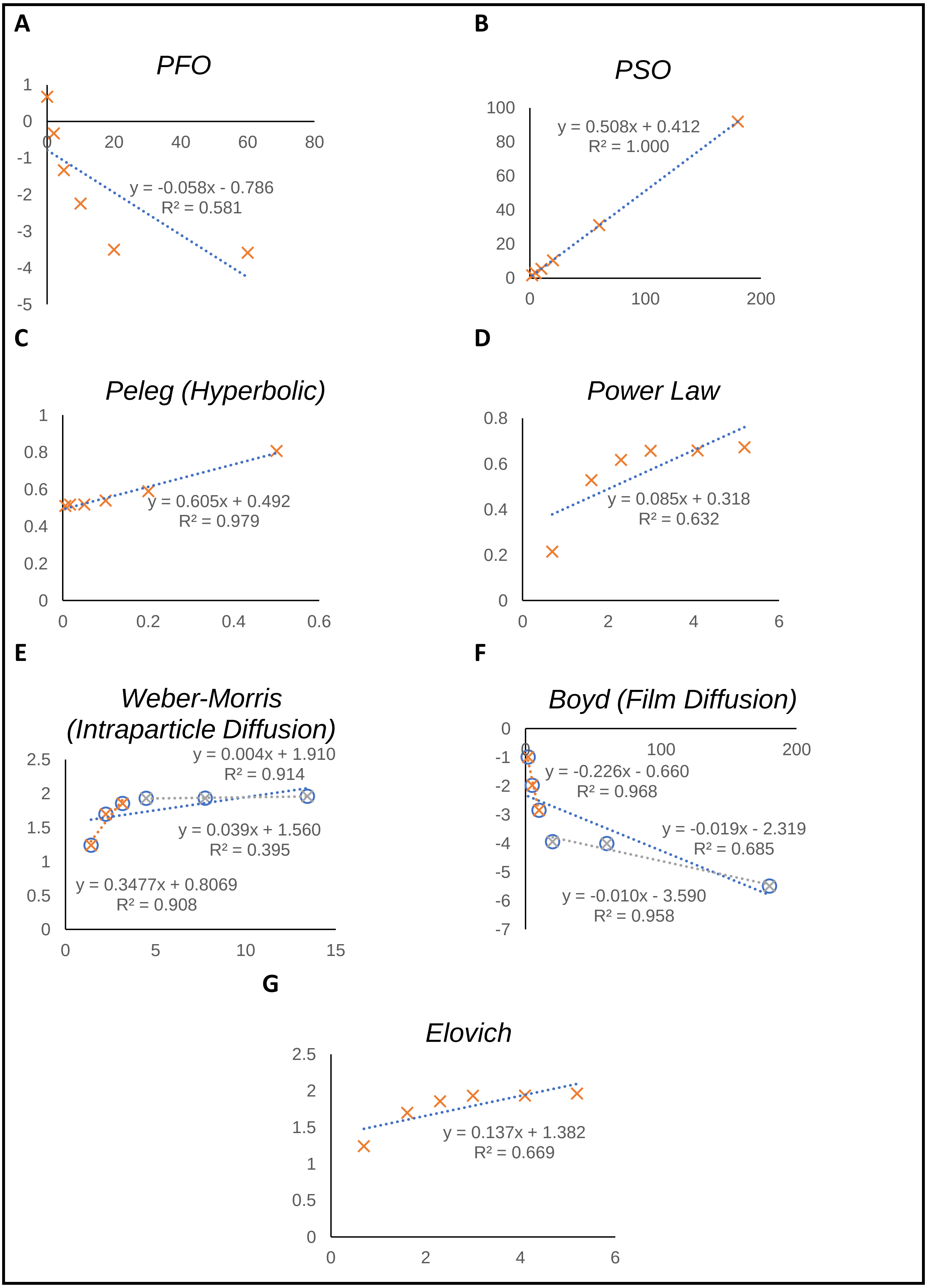

Table S9: *MS/MS Targeted characterization of signature compounds. Ionization polarity, exact mass and first five fragmentations (Da) with related DP and CE (V).*

**Compound**		**Exact Mass**	**Ionization Mode**	**Detected Mass***	**DP**	**Fragments** (number and CE)									
**Name**	**Formula**					**1**	**CE**	**2**	**CE**	**3**	**CE**	**4**	**CE**	**5**	**CE**
Quercetin	$C_{15}H_{10}O_7$	302.0427	NEG	301.0353	-105	151.0046	-30	178.9992	-25	121.0299	-40	65.0037	-70	107.0146	-40
Kaemferol	$C_{15}H_{10}O_6$	286.0477	NEG	285.0429	-150	93.0352	-45	117.0353	-60	185.0621	-40	229.0523	-35	187.0413	-40
Caffeic acid	$C_9H_8O_4$	180.0427	NEG	179.0350	-150	135.0469	-25	134.0388	-35	107.0517	-30	89.0408	-45	79.0565	-35
Chlorogenic acid	$C_{16}H_{18}O_9$	354.0951	NEG	353.0894	-100	173.0472	-25	179.0367	-25	135.0465	-45	191.0578	-35	93.0355	-55
Ferulic acid	$C_{10}H_{10}O_4$	194.0579	NEG	193.0518	-30	134.0389	-20	178.029	-20	149.0625	-20	133.0306	-35	102.9344	-20
Quinic acid	$C_7H_{12}O_6$	192.0634	NEG	191.0548	-120	85.0299	-35	93.0350	-30	59.0143	-30	87.0091	-35	127.0408	-30
Protocatecuic acid	$C_7H_6O_4$	154.0266	NEG	153.0184	-85	109.0304	-20	108.0224	-35	91.0193	-35	81.0347	-25	109.0629	-20
Ellagic Acid	$C_{14}H_6O_8$	302.0063	NEG	300.9970	-90	145.0286	-50	283.9948	-40	229.0129	-35	173.0234	-45	201.0185	-45
Gallic	$C_7H_6O_5$	170.0215	NEG	169.0135	-90	125.0256	-20	79.0195	-35	124.0174	-35	51.0242	-45	81.0350	-25
Catechin	$C_{15}H_{14}O_6$	290.079	NEG	289.0700	-115	245.0831	-25	109.0301	-45	123.0456	-45	203.0723	-30	125.0247	-30
Epicatechin	$C_{15}H_{14}O_6$	290.079	POS	291.0846	+95	139.0365	+25	123.0418	+45	51.0216	+110	165.0519	+20	147.0414	+35
Epigallocatechin	$C_{15}H_{14}O_7$	306.074	POS	307.0810	+90	139.0368	+25	53.0008	+95	163.0363	+30	181.0468	+20	289.0667	+15
Procyanidin B1	$C_{30}H_{26}O_{12}$	578.1424	POS	579.1501	+120	427.098	+25	127.0374	+40	409.088	+30	289.0679	+25	291.0837	+20
Procyanidin B2	$C_{30}H_{26}O_{12}$	578.1424	NEG	577.1316	-130	425.0878	-25	407.0777	-30	289.0718	-35	125.0245	-45	451.1036	-30

*Detected Mass: ±0.0005

Table S10: *Guided MRM HR Infusion information for principal identified compounds, Declustering potential (DP) and collision energy (CE) optimization. General HRMS set-up and conditions for: A. quercetin; B. kaemferol; C. caffeic acid; D. chlorogenic acid; E. ferulic acid; F. quinic acid; G. protocatecuic acid; H. ellagic acid: I. gallic acid; J. catechin; K. epicatechin; L. epigallocatechin; M. procyanidin B1; N. procyanidin B2.*

	Polarity	***Negative***	***Positive***
**General HRMS Set-up**	Initial Conditions Pass TOF Start mass (Da):	100	100
	TOF Stop mass (Da):	1000	1000
	Declustering Potential, DP (V):	-80	80
	Accumulation Time (sec):	0.25	0.25
	Collision Energy, CE (V):	-10	10
	Spray voltage (V):	-4500	5500
	Curtain gas (psi):	45	45
	Ion source gas 1 (psi):	35	35
	Ion source gas 2 (psi):	55	55
	Temperature (°C)	450	350

A. Quercetin

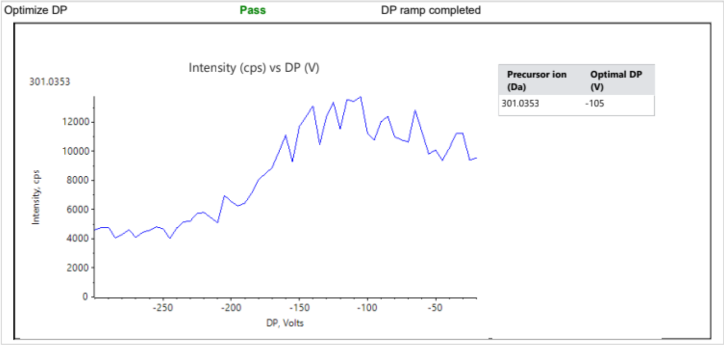

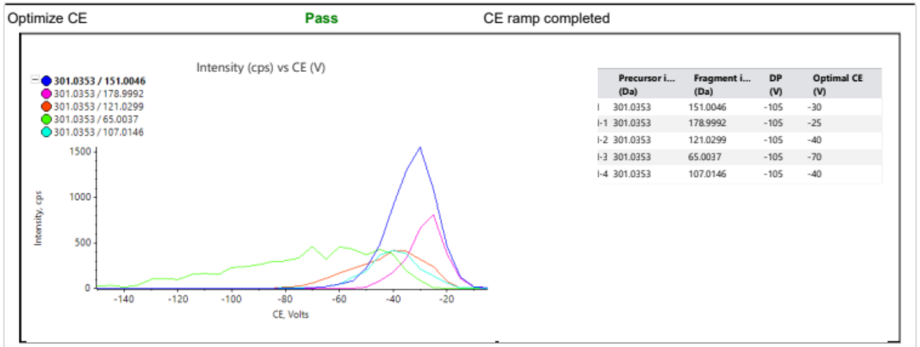

B. Kaemferol

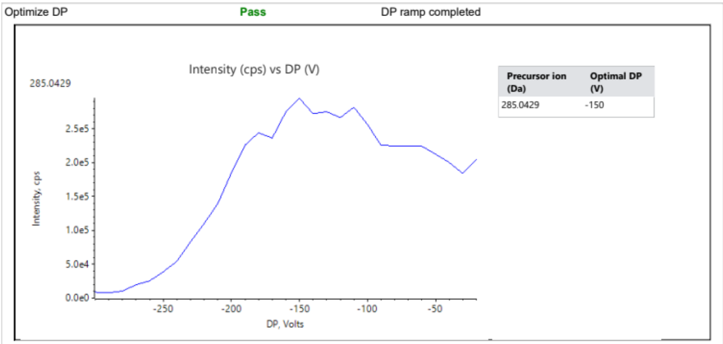

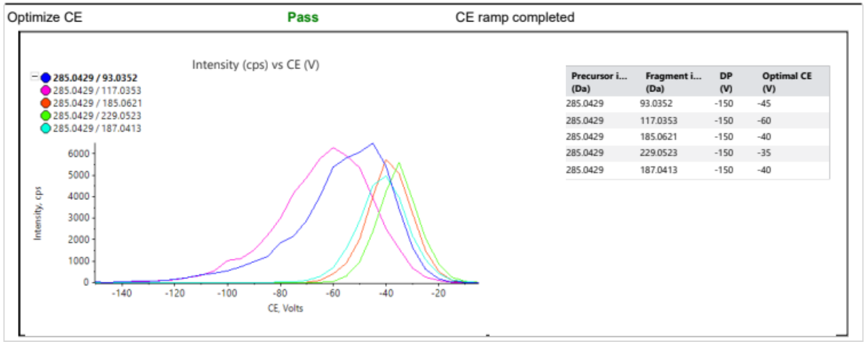

C. Caffeic acid

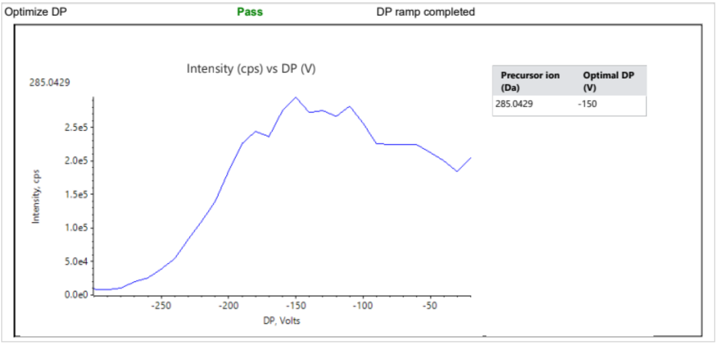

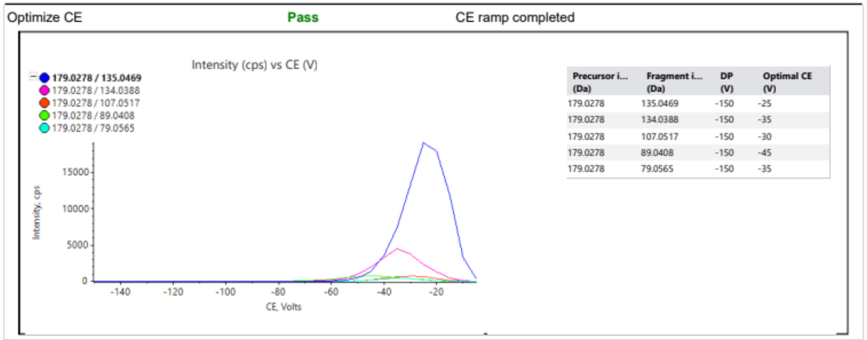

D. Chlorogenic acid

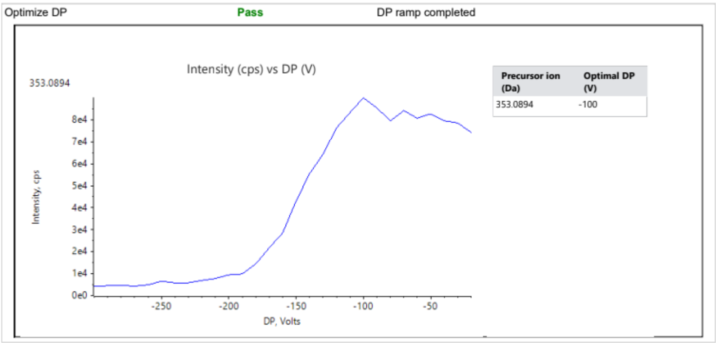

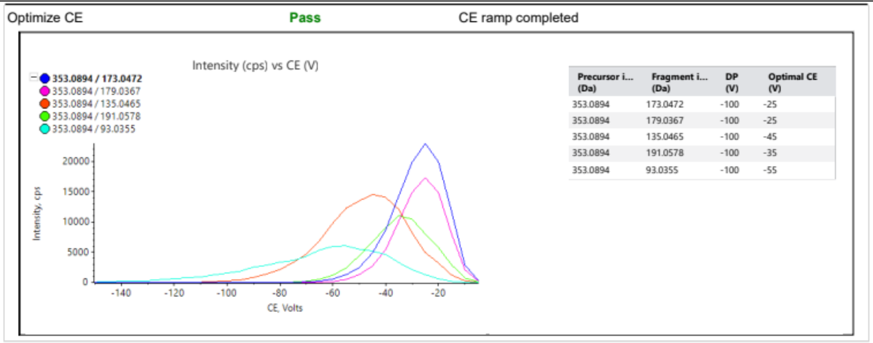

E. Ferulic acid

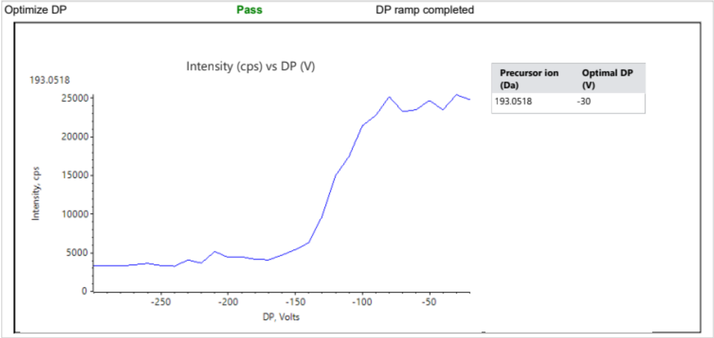

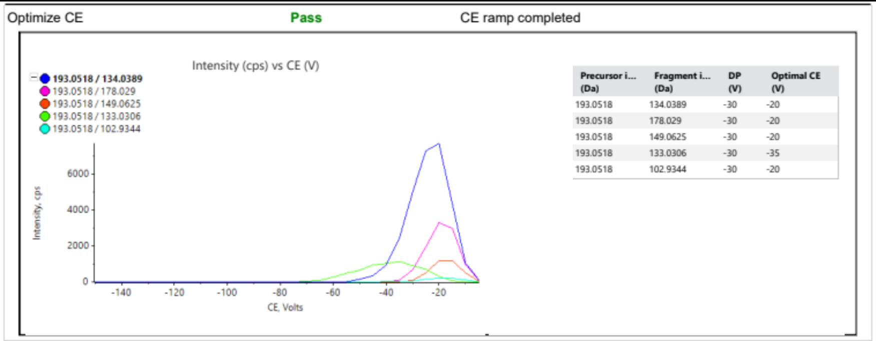

F. Quinic acid

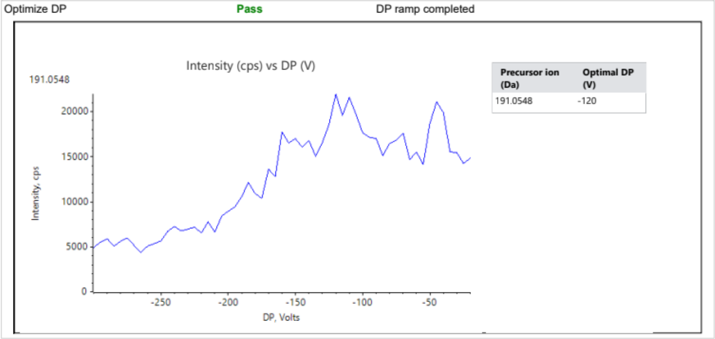

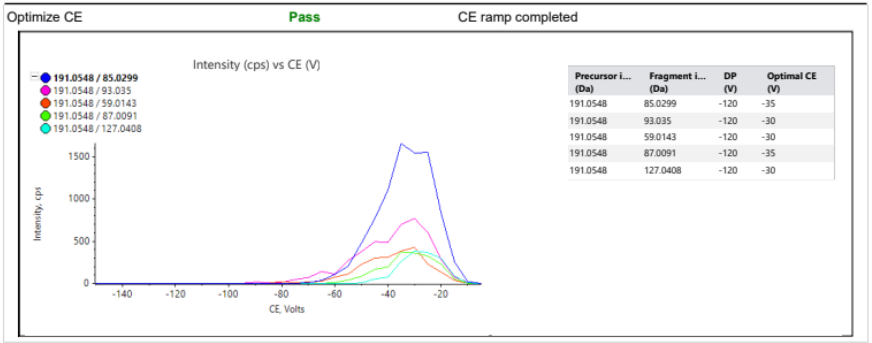

G. Protocatecuic acid

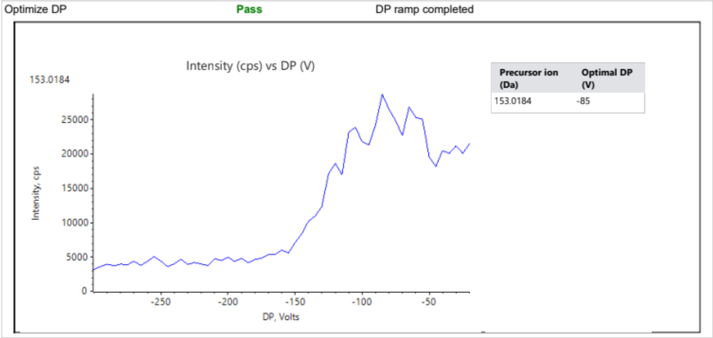

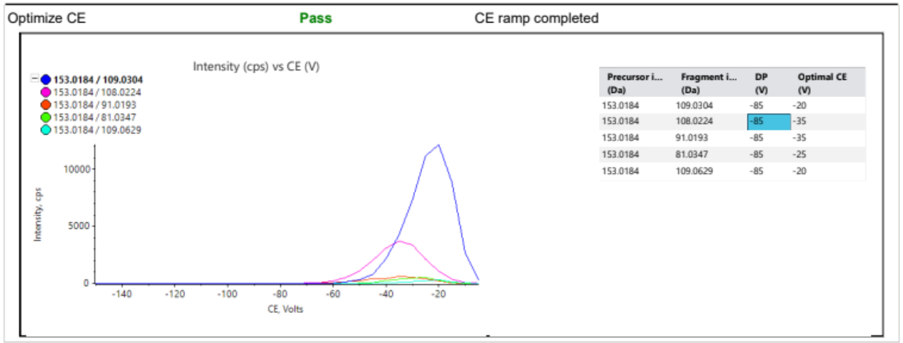

H. Ellagic Acid

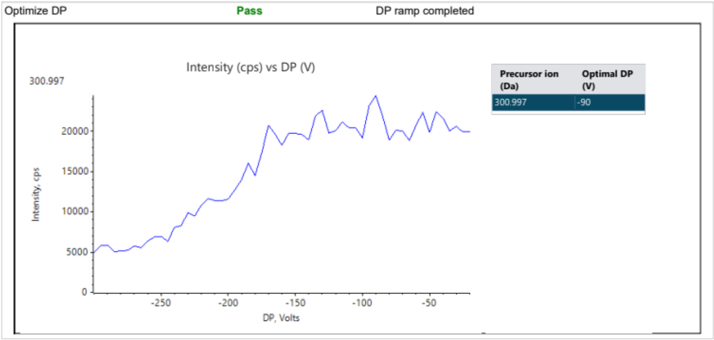

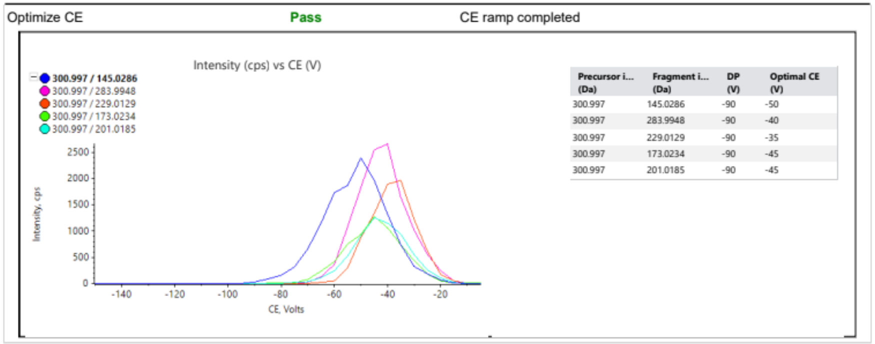

I. Gallic acid

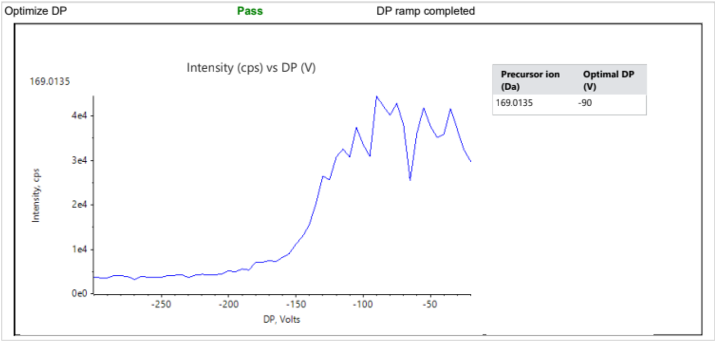

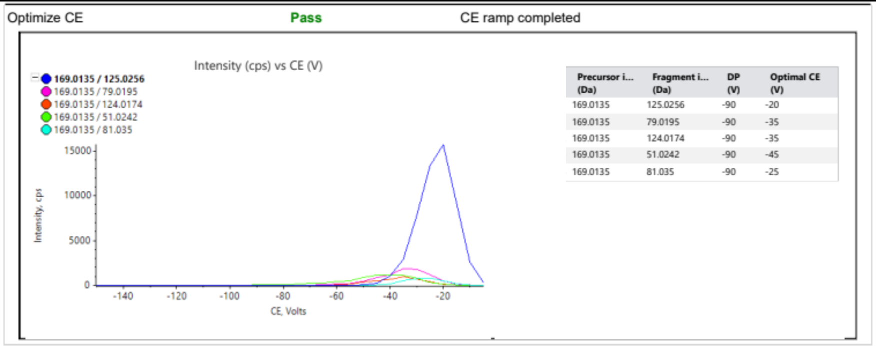

J. Catechin

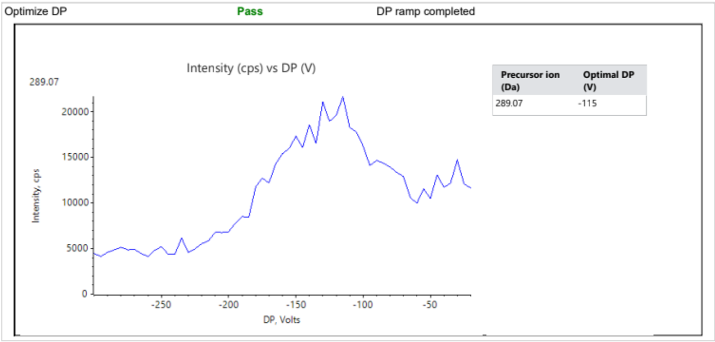

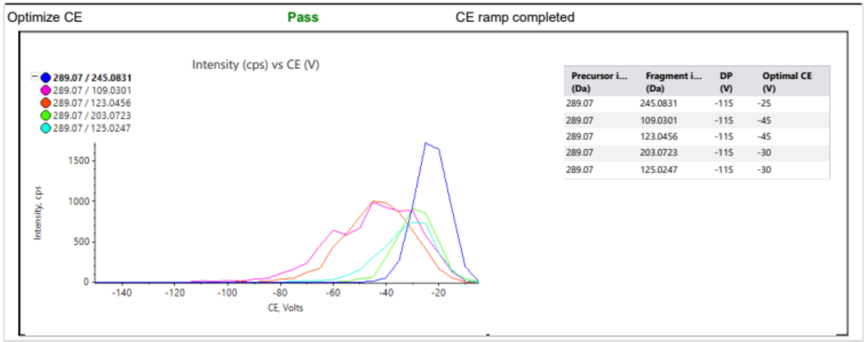

K. Epicatechin

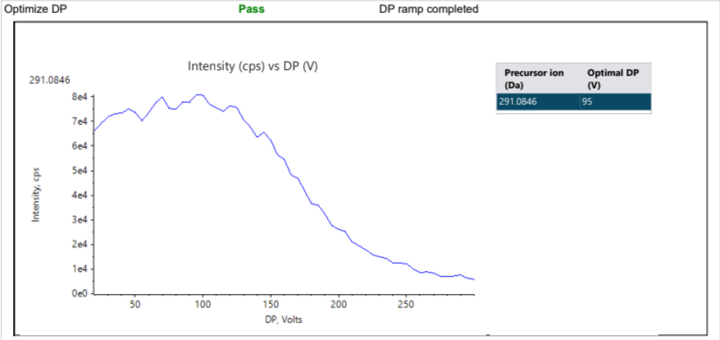

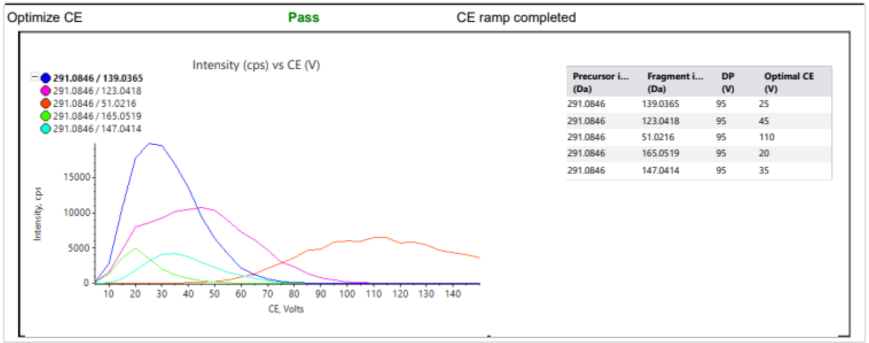

L. Epigallocatechin

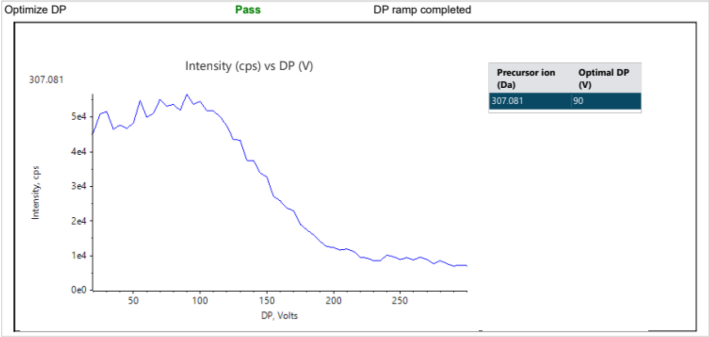

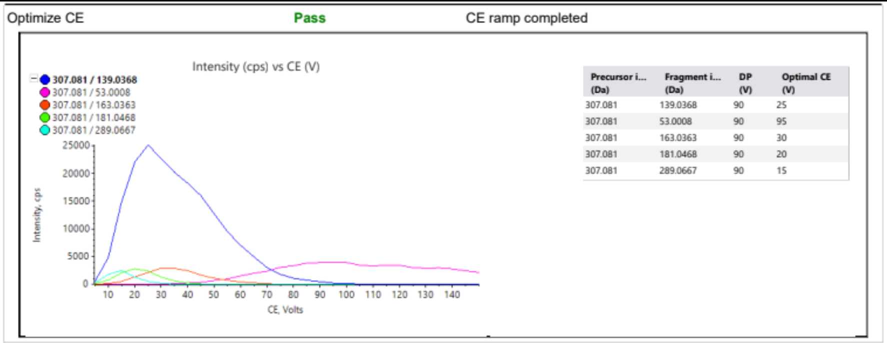

M. Procyanidin B1

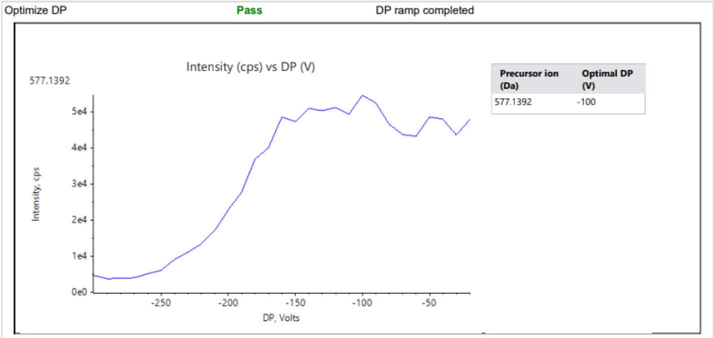

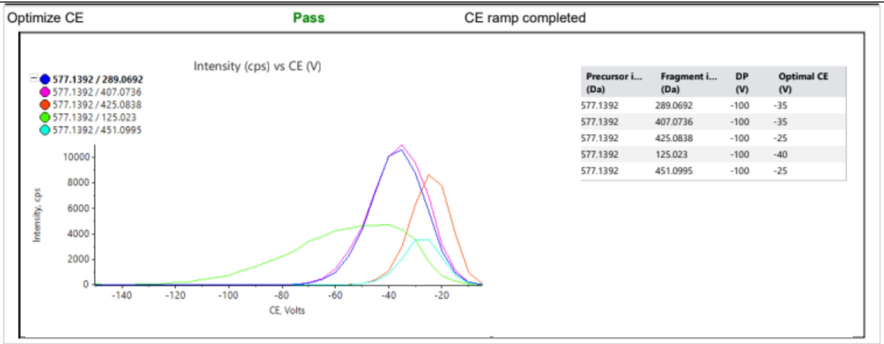

N. Procyanidin B2

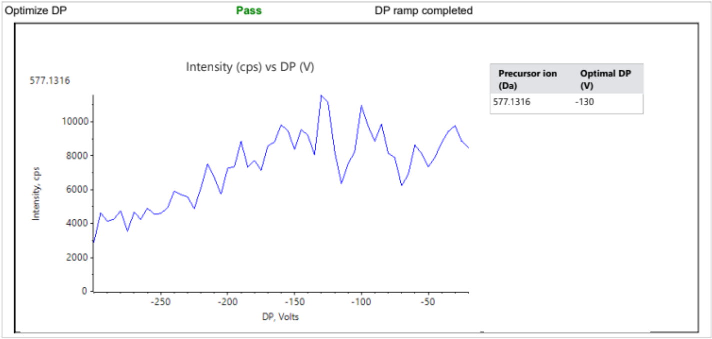

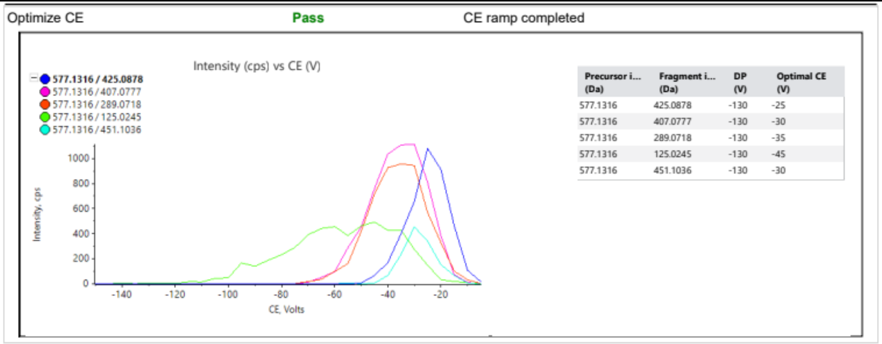

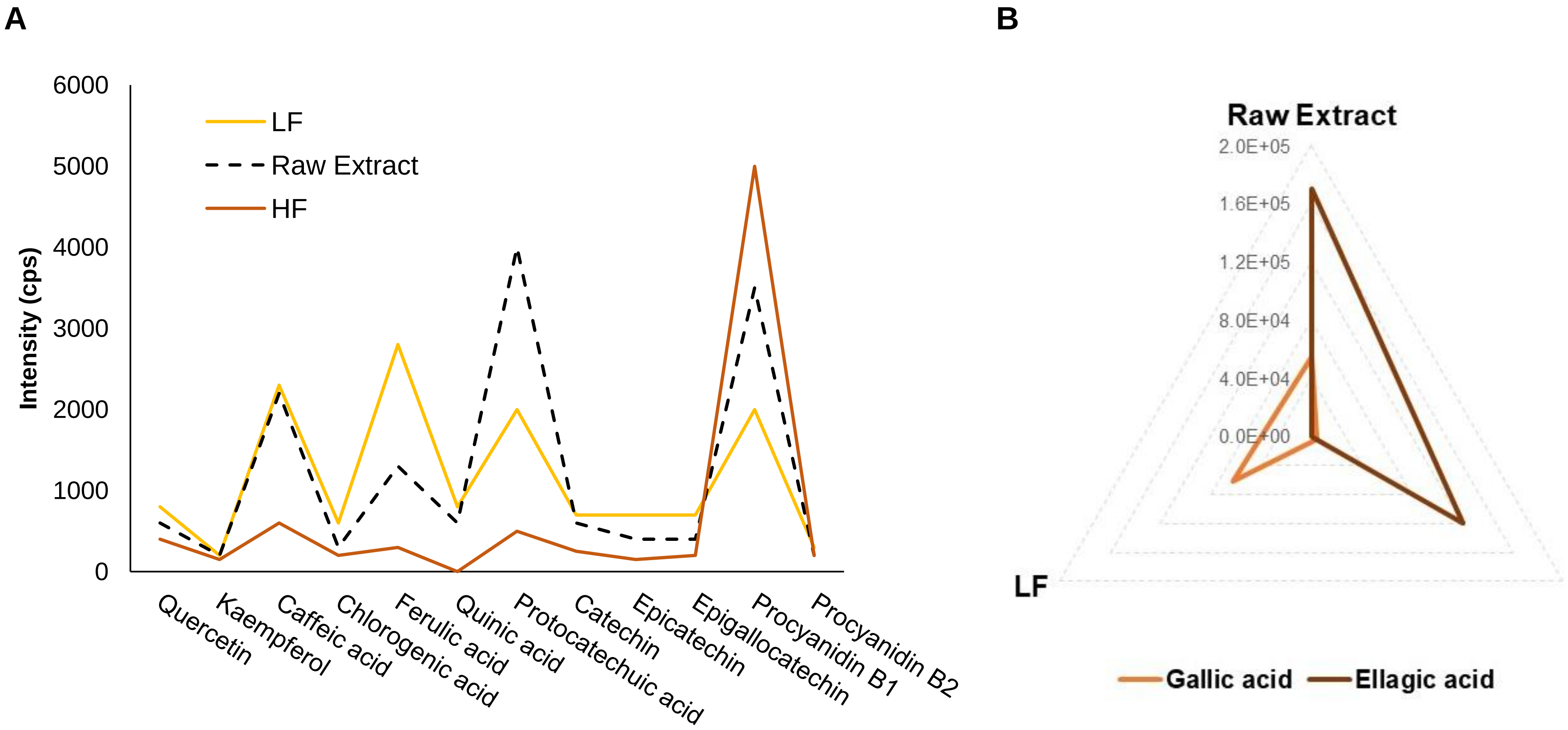

Figure S15*:* *Targeted HRMS characterization of signature compounds for Raw Extract, LF and HF. A: Intensity report for principal identified compounds; B: Direct comparison of gallic and ellagic acid.*

Discussion:

A: Intensity report for principal identified compounds. It worth notice that low molecular-weight compounds underwent to a concentration from Raw Extract to the LF, as expected by the MWCO imposed by membrane filtrations. Procyanidin B1, on the contrary, is retained in the HF, probably due to the matrix effect present during the fractionation.

B: Direct comparison of gallic and ellagic acid. Gallic acid and ellagic acid can be adopted as diagnostic peaks being precursor of hydrolysable tannins. It is interesting to state that gallic acid follow the trend of low molecular-weight molecules, as reported in the previous Figure. On the contrary, ellagic acid seems to be mainly retained in the HF, with minor losses in comparison with the raw extract. Further studies are required to shed light on this behaviour, in particular in relation with the tannin population.

Notes and references

[1] C. Valle, G. Grillo, E. C. Gaudino, P. Ponsetto, R. Mazzoli, G. Bonavita, P. Vitale, E. Pessione, E. Garcia-Moruno, A. Costantini, G. Cravotto, S. Tabasso, "Grape Stalks Valorization towards Circular Economy: A Cascade Biorefinery Strategy" *Chem Sus Chem* **2025**, e202402536. DOI: 10.1002/cssc.202402536.

[2] T. Chen; Z. Wu, W. Wei, Y. Xie, X.A. Wang, M. Niu, Q. Wei, J. Rao, "Hybrid composites of polyvinyl alcohol (PVA)/Si–Al for improving the properties of ultra-low density fiberboard (ULDF)" *RSC Adv.* **2016**, *6*, 20706–20712. DOI: 10.1039/C5RA26868K.

[3] N. Jaipakdee, T. Pongjanyakul, E. Limpongsa, "Preparation and characterization of poly (vinyl alcohol)-poly (vinyl pyrrolidone) mucoadhesive buccal patches for delivery of lidocaine HCL" *Int. J. Appl. Pharm* **2018**, *10*, 115–123. DOI: 0.22159/ijap.2018v10i1.23208

[4] O.N. Tretinnikov, S.A Zagorskaya, "Determination of the degree of crystallinity of poly(vinyl alcohol) by FTIR spectroscopy" *J. Appl. Spectrosc.* **2012**, *79*, 521–526. DOI: 10.1007/s10812-012-9634-y

[5] G. Kovtun, D. Casas, T. Cuberes, "Influence of Glycerol on the Surface Morphology and Crystallinity of Polyvinyl Alcohol Films", *Polymers,* **2024**, 16 (17), 2421. DOI: 10.3390/polym16172421.

[6] A.Ricci, M.-C- Lagel, G. P. Parpinello, A. Pizzi, P. A. Kilmartin, A. Versari, "Spectroscopy analysis of phenolic and sugar patterns in a food grade chestnut tannin", *Food Chem.*, **2016**, *203*, 425-429. DOI: 10.1016/j.foodchem.2016.02.105.

[7] Z.Z. Fu, Y.H., Yao, S.J Guo, K. Wang, Q. Zhang, Q. Fu, "Effect of Plasticization on Stretching Stability of Poly(Vinyl Alcohol) Films: A Case Study Using Glycerol and Water" *Macromol. Rapid Commun.* **2023**, *44*, 2200296. DOI: 10.1002/marc.202200296

[8] N. Stachowiak, J. Kowalonek, J. Kozlowska, "Effect of plasticizer and surfactant on the properties of poly(vinyl alcohol)/chitosan films" *Int. J. Biol. Macromol.* **2020**, *164*, 2100–2107. DOI: 10.1016/j.ijbiomac.2020.08.001
